# Supplementary material for: Aromatic Decoration Determines the Formation of Anthocyanic Vacuolar Inclusions
Source: Curr Biol. 2017 Apr 3;27(7):945–57. doi: 10.1016/j.cub.2017.02.027 (PMC5387179; doi:10.1016/j.cub.2017.02.027)
Supplement: Document S1. Figures S1–S5, Tables S1 and S2, and Supplemental Experimental Procedures [file mmc1.pdf]

Current Biology, Volume *27*

Supplemental Information

Aromatic Decoration Determines the Formation of Anthocyanic Vacuolar Inclusions

Kalyani Kallam, Ingo Appelhagen, Jie Luo, Nick Albert, Huaibi Zhang, Simon Deroles, Lionel Hill, Kim Findlay, Øyvind M. Andersen, Kevin Davies, and Cathie Martin

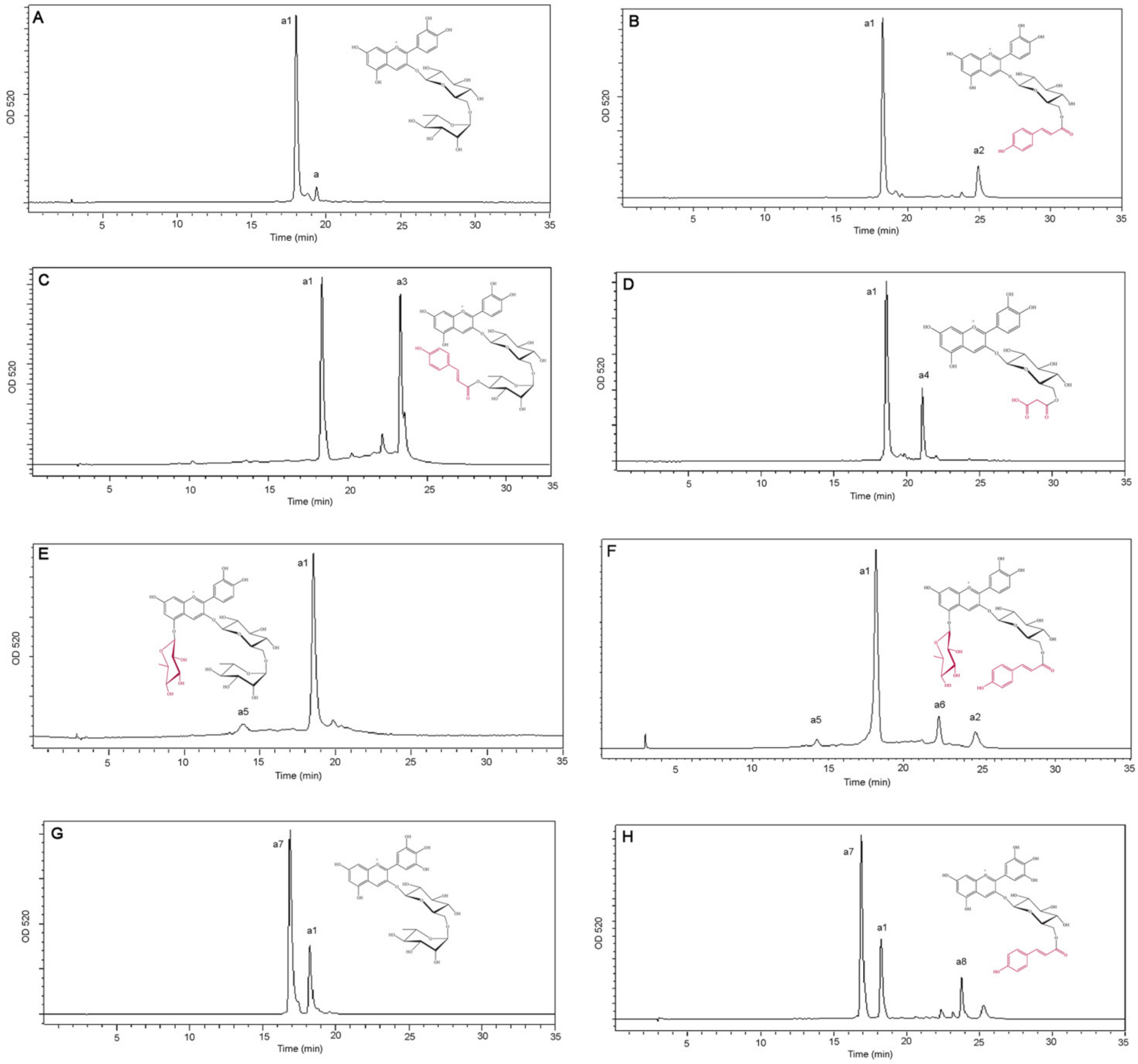

Figure S1: HPLC separation of anthocyanins in different cell lines (Related to Figure 1 and Figure 2)

HPLC chromatograms of leaf extracts in methanol and recorded at 520 nm (**A**) *Del/Ros1* (**B**) *Del/Ros1/At3AT* (**C**) *Del/Ros1/Sl3AT* (**D**) *Del/Ros1/Ci3MAT* (**E**) *Del/Ros1/At5GT* (**F**) *Del/Ros1/At3AT/At5GT* (**G**) *Del/Ros1/PhF3'5'H* (**H**) *Del/Ros1/At3AT/PhF3'5'H*. The methanol extracts of anthocyanins were run on the Surveyor HPLC attached to DecaXPplus ion trap MS (Thermo) and separation was on a 100×2mm 3μm Luna C18(2) column (Phenomenex). Anthocyanin peaks are labelled with numbered alphabets and identity of the new compounds are provided next to the peaks. a=pelargonidin 3-*O*-rutinoside; a1=cyanidin 3-*O*-rutinoside; a2= cyanidin 3-*O*-(6''-*O*-(coumaroyl) glucoside); a3=cyanidin 3-*O*-(4'''-*O*-(coumaroyl) rutinoside); a4=cyanidin 3-*O*-(6''-*O*-(malonyl) glucoside); a5=cyanidin 3-*O*-rutinoside, 5-*O*-glucoside; a6=cyanidin 3-*O*-(6''-*O*-(coumaroyl) glucoside)-5-*O*-glucoside; a7=delphinidin 3-*O*-rutinoside; a8=delphinidin 3-*O*-(6''-*O*-(coumaroyl) glucoside)

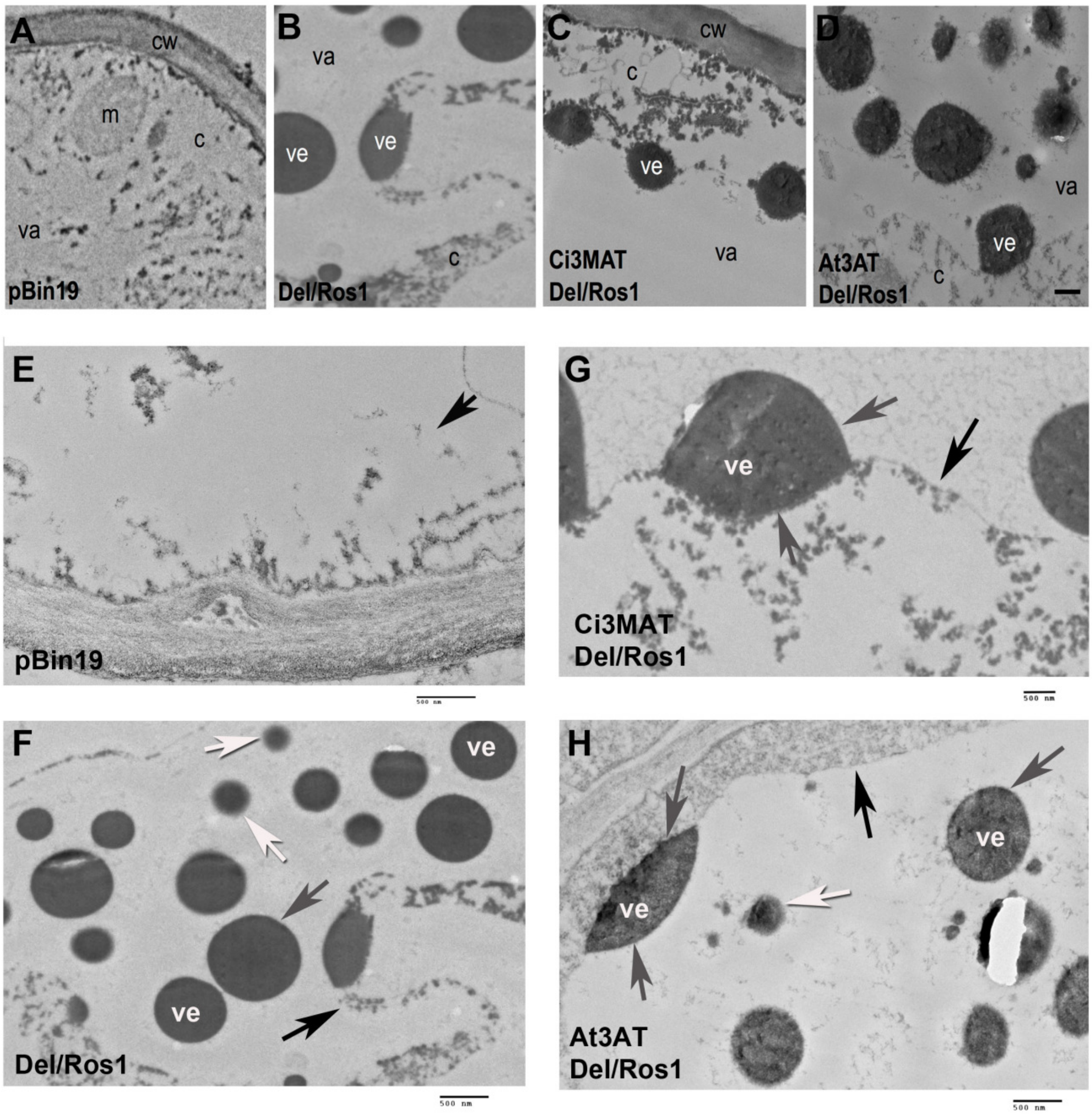

Figure S2. Transmission electron micrographs of callus culture cells (Related to Figure 2)

(**A**) Wild type tobacco cell transformed with an empty pBin19 binary vector alone. (**B**) *Del/Ros1* making cyanidin 3-*O*-rutinoside only, producing no AVIs, (**C**) *Del/Ros1/Ci3MAT* making cyanidin 3-*O*-rutinoside and cyanidin 3-*O*-(6''-*O*-(malonyl) glucoside), producing no AVIs. (**D**) *Del/Ros1/At3AT* making cyanidin 3-*O*-rutinoside and cyanidin 3-*O*-(6''-*O*-(coumaroyl) glucoside) and producing AVIs. c= cytoplasm, va = vacuole, cw = cell wall, ve = anthocyanin-filled vesicle, m = mitochondrion. Scale bar indicates 0.5 µm. (**E**) Higher magnification image of wild type tobacco cell transformed with an empty pBin19 binary vector alone (**F**) Higher magnification image of *Del/Ros1* making cyanidin 3-*O*-rutinoside only, producing no AVIs (**G**) Higher magnification image of *Del/Ros1/Ci3MAT* making cyanidin 3-*O*-rutinoside and cyanidin 3-*O*-(malonyl) glucoside, producing no AVIs (**H**) *Del/Ros1/At3AT* making cyanidin 3-*O*-rutinoside and cyanidin 3-*O*-(coumaroyl) glucoside and producing AVIs. (**E-H**) ve shows anthocyanin filled vesicle, black arrows indicate tonoplast, grey arrows indicate membranes around vesicles and white arrows indicate vacuolar vesicles around which membranes are disintegrating; scale bars show 500 nm.

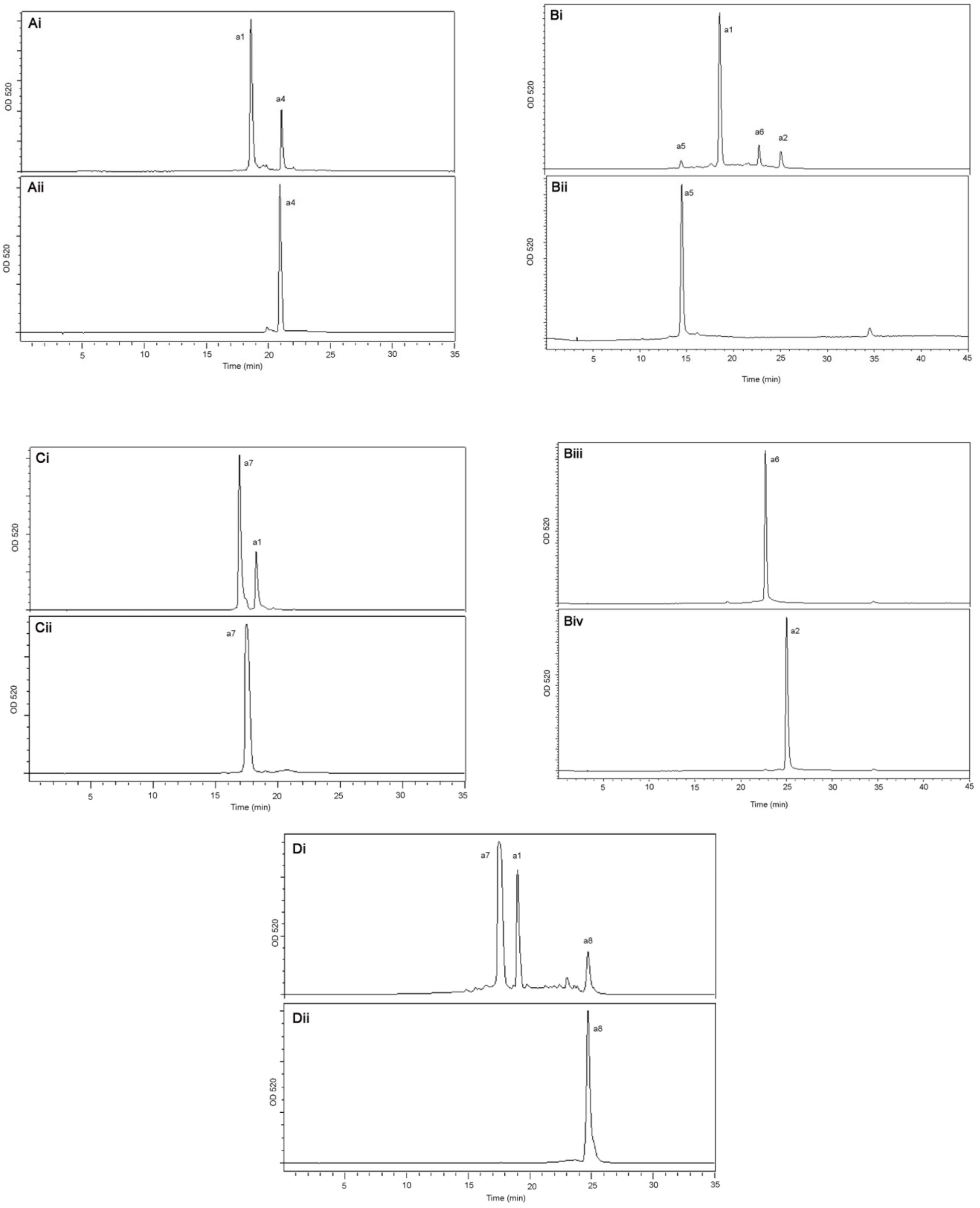

Figure S3. Analytical HPLC analysis of anthocyanins purified from tobacco (Related to Figure 3)

Chromatograms of purified anthocyanins in methanol as studied through HPLC and recorded at 520 nm (**Ai**) Total anthocyanins from leaf extracts of *Del/Ros1/Ci3MAT* and (**Aii**) purified anthocyanin, peak-a1; cyanidin 3-*O*-rutinoside and peak-a4; cyanidin 3-*O*-(malonyl) glucoside (**Bi**) Extracts of total anthocyanins from *Del/Ros1/At3AT/At5GT* line and (**Bii, Biii, Biv**) purified anthocyanins, peak-a5; cyanidin 3-*O*-rutinoside-5-*O*-glucoside, peak-a6; cyanidin 3-*O*-(6''-*O*-(coumaroyl) glucoside)-5-*O*-glucoside, peak-a2; cyanidin 3-*O*-(6''-*O*-(coumaroyl) glucoside) (**Ci**) HPLC chromatogram of total anthocyanins from leaf extracts of *Del/Ros1/PhF3'5'H* and (**Cii**) purified anthocyanin, peak-a1; cyanidin 3-*O*-rutinoside and peak-a7; delphinidin 3-*O*-rutinoside (**Di**) HPLC chromatogram of total anthocyanins from leaf extracts of *Del/Ros1/PhF3'5'H/At3AT* and (**Dii**) purified anthocyanin, peak-a1; cyanidin 3-*O*-rutinoside, peak-a7; delphinidin 3-*O*-rutinoside, peak-a8; delphinidin 3-*O*-(6''-*O*-(coumaroyl) glucoside). Further confirmation of purity of anthocyanins was achieved by mass fragmentation (LC-MS) and chromatogram studies at 280 nm.

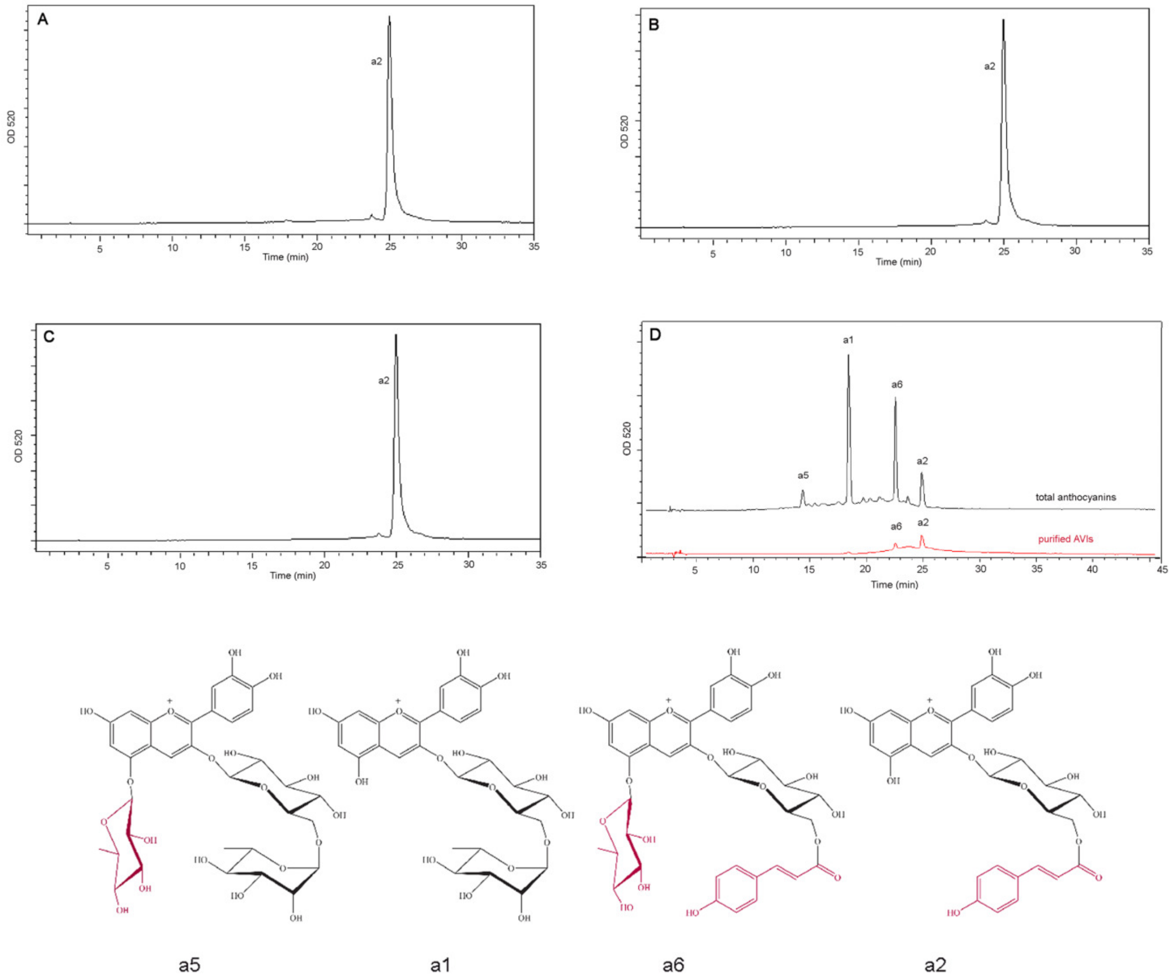

Figure S4. Purification and analysis of *in vitro* anthocyanin precipitates (Related to Figure 3)

(A-C) A sample of purified cyanidin 3-*O*-(6''-*O*-(coumaroyl)glucoside) in 80% methanol was separated by HPLC (**A**). Cyanidin 3-*O*-(6''-*O*-(coumaroyl)glucoside) was added to 0.4 M sodium acetate buffer at pH 4.5 (**B**) or McIlvaine's buffer at pH 7.0 (**C**) and *in vitro* precipitates were purified by centrifugation and washing in buffer, then re-dissolved in 80% methanol and separated by HPLC. The peaks from the precipitates ran at exactly the same position (just over 25 minutes) as the cyanidin 3-*O*-(6''-*O*-(coumaroyl)glucoside) in solution.

(D) Purification of precipitates from anthocyanin mixtures from *Del/Ros/At3AT/At5GT* line. HPLC profiles of total anthocyanins (top in black) and anthocyanins extracted from precipitates formed *in vitro* by addition of 0.4 M sodium acetate buffer, pH 4.5 (bottom in red). Peak-a5; cyanidin 3-*O*-glucoside-5-*O*-glucoside, peak-a1; cyanidin 3-*O*-rutinoside. Cyanidin 3-*O*-(6''-*O*-(coumaroyl) glucoside)-5-*O*-glucoside (peak a6) and cyanidin 3-*O*-(6''-*O*-(coumaroyl)glucoside) (peak a2) were detected in AVIs with the major anthocyanin being cyanidin 3-*O*-(6''-*O*-(coumaroyl)glucoside) (peak a2). Chemical structures of the anthocyanins corresponding to different peaks (a1, a2, a5 and a6) are shown below the HPLC profiles.

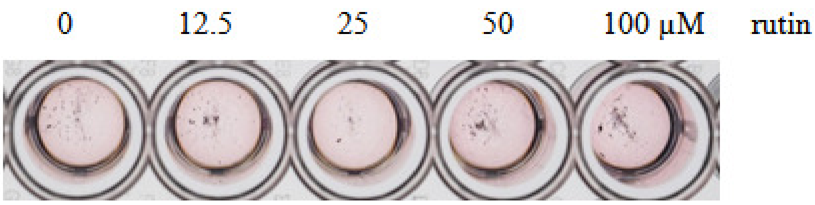

Figure S5. Effects of flavonols on anthocyanin precipitation *in vitro* (Related to Figure 2)

In vitro precipitation of cyanidin 3-*O*-(6''-*O*-(coumaroyl)glucoside) from tobacco in 0.1 M sodium citrate buffer at pH 4.5 with increasing concentrations of the flavonol glycoside, rutin, added prior to adding the anthocyanin at 50 µM.

Transgenic line	Peak label	Rt (min)	ESI-MS (m/z)	MS/MS fragments	Compound
Del/Ros1	a1<br>a	18.65<br>19.9	595<br>579.1	449 [Cy+Glc]$^{+}$ 287 [Cy]$^{+}$<br>433 [Pg+Glc]$^{+}$ 271.3 [Pg]$^{+}$	cyanidin 3-*O*- rutinoside<br>pelargonidin 3-*O*-rutinoside
Del/Ros1/<br>At3AT	a1<br>a2	18.48<br>25.04	595<br>595	449 [Cy+Glc]$^{+}$ 287 [Cy]$^{+}$<br>449 [Cy+Glc]$^{+}$ 287 [Cy]$^{+}$	cyanidin 3-*O*- rutinoside<br>cyanidin 3-*O*-(6″-*O*- (coumaroyl) glucoside)
Del/Ros1/<br>Sl3AT	a1<br>a3	18.37<br>23.33	595.1<br>741.2	449 [Cy+Glc]$^{+}$ 287 [Cy]$^{+}$<br>594.9 [Cy+Rha]$^{+}$ 449 [Cy+Glc]$^{+}$ 287 [Cy]$^{+}$	cyanidin 3-*O*- rutinoside<br>cyanidin 3-*O*-(6″-*O*- (coumaroyl) rutinoside)
Del/Ros1/<br>Ch3MAT	a1<br>a4	17.97<br>20.6	595<br>535	449 [Cy+Glc]$^{+}$ 287 [Cy]$^{+}$<br>449 [Cy+Glc]$^{+}$ 287 [Cy]$^{+}$	cyanidin 3-*O*- rutinoside<br>cyanidin 3-*O*-(6″ -*O*-(malonyl) glucoside)
Del/Ros1/<br>At5GT	a5<br><br>a1	13.9<br><br>18.52	751.1<br><br>595	595 [Cy+Rha]$^{+}$ 449 [Cy+Glc]$^{+}$ 287 [Cy]$^{+}$<br>449 [Cy+Glc]$^{+}$ 287 [Cy]$^{+}$	cyanidin 3-*O*- rutinoside, 5- *O*-glucoside<br><br>cyanidin 3-*O*- rutinoside
Del/Ros1/<br>At3AT/<br>At5GT	a1<br>a5<br><br>a6<br><br>a2	18.45<br>14.26<br><br>22.31<br><br>24.75	595<br>751.1<br><br>757<br><br>595	449 [Cy+Glc]$^{+}$ 287 [Cy]$^{+}$<br>595 [Cy+Rha]$^{+}$ 449 [Cy+Glc]$^{+}$ 287 [Cy]$^{+}$<br>595 [Cy+Glc+Cou]$^{+}$ 449 [Cy+Glc]$^{+}$ 287 [Cy]$^{+}$<br>449 [Cy+Glc]$^{+}$ 287 [Cy]$^{+}$	cyanidin 3-*O*- rutinoside<br>cyanidin 3-*O*- rutinoside-5-glucoside<br><br>cyanidin 3-*O*-(6″-*O*- (coumaroyl) glucoside)-5-*O*-glucoside<br>cyanidin 3-*O*-(6"-*O*-(coumaroyl) glucoside)
Del/Ros1/<br>PhF3'5'H	a7<br>a1	16.78<br>18.19	611.1<br>595	464.97 [Dp+Glc]$^{+}$ 303.1 [Dp]$^{+}$<br>449 [Cy+Glc]$^{+}$ 287 [Cy]$^{+}$	delphinidin 3-*O*-rutinoside<br>cyanidin 3-*O*- rutinoside
Del/Ros1/<br>PhF3'5'H/<br>At3AT	a7<br>a1<br>a8<br>a2	16.9<br>18.27<br>23.79<br>25.29	611.1<br>595<br>611.14<br>595	464.97 [Dp+Glc]$^{+}$ 303.1 [Dp]$^{+}$<br>449 [Cy+Glc]$^{+}$ 287 [Cy]$^{+}$<br>464.97 [Dp+Glc]$^{+}$ 303.1 [Dp]$^{+}$<br>449 [Cy+Glc]$^{+}$ 287 [Cy]$^{+}$	delphinidin 3-*O*-rutinoside<br>cyanidin 3-*O*- rutinoside<br>delphinidin 3-*O*-(6″ -*O*-(coumaroyl) glucoside)<br>cyanidin 3-*O*-(6″ -*O*-(coumaroyl) glucoside)

Table S1. Characterisation of anthocyanins engineered in tobacco (Related to Figure 3 and Figure S1)

Characteristics of anthocyanins from Figure S1; their molecular ions and corresponding fragments together with the identity of each compound are tabulated. ESI-MS; Electro-spray ionization mass spectra, *m/z*; molecular mass of compound. The molecular mass and fragmentation patterns of cyanidin 3-*O*-rutinoside, 5-*O*-glucoside and cyanidin 3-*O*-(6"-*O*-(coumaroyl)glucoside)-5-*O*-glucoside being the same, further NMR confirmation of cyanidin 3-*O*-(6"-*O*-(coumaroyl)glucoside)-5-*O*-glucoside was performed with purified compounds (data not provided). Rt, retention time; Cy; cyanidin, Dp; delphinidin, Pg; pelargonidin, Rha; rhamnose, Glc; glucose

Peak	ESI-MS (m/z)	Mass accuracy (ppm)	Formula	Compound
1	[M]$^{+}$ 1337.3655	3.07	$C_{59}H_{69}O_{35}^{+}$	Violdelphin+Glc
2	[M]$^{+}$ 1175.3099	1.11	$C_{53}H_{59}O_{30}^{+}$	Violdelphin
3	[M]$^{+}$ 1029.2519	1.17	$C_{47}H_{49}O_{26}^{+}$	Violdelphin-Rha
4	[M]$^{+}$ 1901.5198<br>[M+K]$^{2+}$ 970.2327	5.52<br>3.71	$C_{85}H_{97}O_{49}^{+}$	Cyanodelphin

Table S2. Characterisation of anthocyanins in delphinium flowers (Related to Figure 5)

IT-ToF peak identification of violdelphin and cyanodelphin are tabulated. ESI-MS; Electro-spray ionization mass spectra, *m/z*; molecular mass of compound.

Supplemental Experimental Procedures
Generation of Plant Material and Growth Conditions

A full length gene, encoding anthocyanin UDP-glucoronosyl/UDP-glucosyl transferase (GeneBank accession AT4G14090 [S1]), a full length cDNA of At1g03940 encoding an anthocyanidin 3-glucoside acyl CoA transferase (At3AT1 [S2]), a full length cDNA (EU979541) encoding anthocyanin 3-rutinoside hydroxycinnamoyl CoA transferase from Solanum lycopersicum cv. MicroTom (Sl3AT1 [S3, S4]), a cDNA encoding flavonoid 3'5' hydroxylase from Petunia hybrida (PhF3'5'H [S5]; CAA80265), and a cDNA from Chrysanthemum indicum, encoding anthocyanidin 3-*O*-glucoside-malonyltransferase Ci3MaT1 (GeneBank accession AY298809 [S6]), amplified from cDNA of dark red (maroon) chrysanthemum inflorescence were cloned into pJAM1502 [S2]. Gene specific primers having Gateway™ recombination sequences; attB1 and attB2 used to amplify each gene sequence are given at the end of this section. Wild type and transgenic tobacco plants were grown at 23- 25°C under 16 h light/8 h dark cycle. Callus from young and healthy transgenic leaf material of genotyped plants was induced on callus induction medium (MS agar (0.8% w/v) supplemented with kanamycin 100 mgL^{-1}, kinetin 0.5 mgL^{-1}, 2,4 D 1.0 mgL^{-1} and NAA 0.5 mgL^{-1}). The plates were incubated in a growth room at 23-25°C under 16 h light/8 h dark or in complete darkness at 23-25°C. Callus formation was better when maintained in darkness, and this condition was applied for all the cultures. Sub-culturing was performed every 15 d for 45 d, until sufficient callus was produced for suspension culture formation.

Anthocyanin extraction and semi-purification

Flower petals from which anthocyanins were to be extracted were ground to a fine powder. Extraction solvent, (70% methanol with 0.1 % HCl, 40 μLmg^{-1}) was added to the samples, which were vortexed thoroughly and sonicated in a water bath for 10 min. Extracts were processed immediately by centrifugation at 12,000 rpm for 10 min at 4°C. Supernatants were collected and stored at -20°C before being analysed. Anthocyanins from finely ground leaf material were extracted using acidified water (0.01% HCl). Two steps of extraction were followed by addition of an equal amount of chloroform at 4°C to remove the chlorophyll. The supernatant was collected the following day and rotary evaporated under vacuum at 30-40°C until any residual chloroform had evaporated. Anthocyanins in acidified water were purified [S7] by passing through a preconditioned C18 mini cartridge (Sep-Pak Cartridge 500 mg sorbent, Waters Chromatography®). The C18 cartridge was initially conditioned by passing over two column volumes of methanol followed by three column volumes of acidified deionized milliQ water to remove the remaining methanol. Each sample was loaded onto the column until excess colour had passed through the cartridge. The cartridge was washed with two column volumes of acidified water to remove compounds not adsorbed (sugars, acids) followed by two column volumes of ethyl acetate to remove polyphenolic compounds such as phenolic acids and flavonols. Anthocyanin pigments were eluted with acidified methanol. Methanol was removed from the extract in a rotary evaporator at 40°C under vacuum and the anthocyanins were re-dissolved in acidified milliQ water and stored at -20°C until further use.

Preparative HPLC purification

Anthocyanins were purified using a Gilson Preparative High Pressure Liquid Chromatography (Prep HPLC) System. Partially purified and filtered extracts (filtered using a 0.2 µm polyfluortetraethylene filter, Sartorius) were applied onto a 250 x 21.2 mm C18 column (Phenomenex) fitted with a guard column (Phenomenex Prodigy). The column was pre-equilibrated with water containing 0.5% triflouroacetic acid (TFA). The sample was injected onto the column, developed by a wash with 96% solvent A (0.5% TFA in water) and 4% solvent B (0.5% TFA in 50% acetonitrile (ACN)). Anthocyanins were eluted with a linear gradient of increasing solvent B at a flow rate of 1 mL min^{-1}: from 20% to 40% over 10 min, 40% to 60% over 5 min, 60% to 80% over 13 min, 80% to 100% over 7 min and 100% for 10 min. The elution products were monitored with a UV detector. This protocol was developed for separation of all the compounds. Fractions were collected and residual acetonitrile in the fractions was removed using a rotary evaporator. Acidified water was added to the samples and freeze dried. Freeze dried anthocyanin samples or concentrated samples after rotary evaporation were stored at -80°C in an air tight container or dissolved in the desired solvent for further studies.

HPLC, LC-MS analysis and quantification of anthocyanins

Anthocyanin extracts were run on a 250 x 4.6 mm internal diameter Spherisorb® 5 µm C18 column using Waters HPLC system (Waters, http://www.waters.com). The column was initially equilibrated with 96% solvent A (0.5% TFA) and 4% solvent B (0.5% TFA in 50% ACN) and eluted with a gradient of increasing solvent B at a flow rate of 1 mL min^{-1}. After injection the column was washed with 96% solvent A followed by a linear gradient of solvent B from 4% to 20% in 5 min, 20% to 40% in 10 min, 40% to 60% in 5 min, 60% to 20% in

10 min and 20% to 4% in 5 min. The elution products were monitored with a photo diode array (PDA) detector over the range 200–600 nm. Anthocyanin contents were analysed based on the peaks obtained at various retention times compared to controls and quantified based on the peak areas at 520 nm in the chromatograms. Commercial anthocyanins: cyanidin 3-*O*-rutinoside and cyanidin 3-*O*-glucoside (Extrasynthase, France) were used as standards. Standards (5 mg) were dissolved in methanol with 0.1% HCl and stored at -80°C. The concentration of anthocyanins in the samples was calculated by measuring the absorbance at 520 nm using a Spectra max 340PC384 (Molecular devices) spectrophotometer [S8]. Readings were analysed using Softmax(R) Pro software version 4.8. Purified anthocyanins and their structural decorations were always confirmed by LC/MS using a Thermo Finnigan Surveyor HPLC system (Thermo Scientific, http://www.thermo.com) equipped with a diode array (PDA) detector and a Deca XP plus ion trap mass spectrometer (Thermo Scientific), prior to undertaking new assays. Anthocyanin masses and fragmentation patterns were analysed using Quant browser.

Anthocyanin samples from delphinium sepals were run on a Shimadzu Nexera LC system attached to an IT ToF mass spectrometer. Separation was on a 100×2.1 mm 2.6 μ Kinetex EVO-C18 column (Phenomenex), using a linear gradient of acetonitrile versus 0.1% formic acid from 2% to 10% acetonitrile in 2.5 min, 10% to 30% in 10 min, 30% to 90% in 5.8 min, from 90% to 2% in 4.3 min, with a flow rate of 0.5mL min^{-1}. Positive electrospray MS spectra from m/z 220-2000 were collected with a maximum ion accumulation time of 20 msec and an automatic sensitivity control target of 70% optimum base peak intensity.

Protoplast and AVI isolation

A protocol for protoplast isolation was adapted from several published protocols. Leaf material or flower material was cut into small pieces in protoplast buffer (0.7 M mannitol, 0.25 mM MES, 1% (w/v) cellulase, 0.25% (w/v) macerozyme, 0.5% (w/v) driselase; pH 5.5) and incubated at 30°C for 4-6 h in dark with gentle shaking (30 rpm). The solution was strained through two layers of Mira cloth and a layer of 50 µm cheese cloth. The filtrate was centrifuged at 4°C for 10 min at 100 rpm. The pellet was washed in protoplast buffer without enzymes and suspended in a small amount of protoplast buffer. Ten times the volume of suspended pellet of 0.7 M sucrose was layered on top of the pellet, centrifuged for 15 min at 100 rpm, 4°C. Intact and healthy protoplasts are collected on top of the sucrose solution. Protoplasts were washed twice with the buffer and suspended in a small amount of buffer and stored at -20°C. Freeze-thawed protoplasts were sonicated to break them and layered on a Percoll gradient (obtained by sequential layering of 80%/50%/30%/10% Percoll with protoplast buffer) and centrifuged at 100 rpm for 10-15 min. AVIs were collected from the bottom of the tube prior to analysis.

Confocal microscopy

Images were taken with Leica SP5II and SP8X confocal microscopes equipped with HCX APO L U-V-I 63.0 x 0.90 UV and HC PL APO CS2 63x/1.20 water immersion objectives (both from Leica Microsystems GmbH). Fluorescence was detected by hybrid detectors unless stated otherwise. Emission spectra of anthocyanins were recorded with 3 nm step size and 10 nm bandwidth after excitation at 561 nm with a diode-pumped solid-state laser. Anthocyanins were subsequently detected between 600-650 nm in leaf tissues to avoid detection of chlorophyll auto-fluorescence, and between 665-730 nm in callus cells without chloroplasts. pH measurements in vacuoles were performed as described by Siedel et al., (2005) [S9] with the following modifications. Leaf disks were incubated overnight in 1xMS medium with 20µM of the non-fluorescent dye 6-carboxyfluorescein diacetate (6-CFDA, Sigma-Aldrich) and 0.35‰ (v/v) Triton X-100 (Sigma-Aldrich). Non-specific vacuolar esterases cleave 6-CFDA and release the fluorescent dye 6-carboxyfluorescein (6-CF), which has a pH-dependent ratio of emission intensities at different excitation wavelengths. 6-CF was excited sequentially using the 458 nm and the 488 nm line of an argon laser and detected in the range of 500-530 nm to avoid crosstalk with anthocyanins. A HC PL APO CS2 20x/0.75 air objective (Leica Microsystems GmbH) was used and images were acquired in 12 bit resolution to maximise the dynamic range. Calibrations were performed with a 6-CF standard (Sigma-Aldrich) in buffers with pH-values from pH 4.65 to 7.0 (1/20 dilutions of a 10 mM 6-CF stock in ethanol), whereby the intensities of the laser lines were adjusted to an emission ratio of about 1.0 at pH 6. Fluorescence from 20 epidermal cells was measured and pH values were calculated from a linear regression curve of the 6-CF reference measurements. For membrane stains, cells were incubated for 16 to 20 h in 4 µM FM1-43 (Thermo Scientific) in 1xMS medium containing 3% (w/v) sucrose. Tobacco cells were incubated in 24-well plates and the dye infiltrated into delphinium sepals with a syringe. FM1-43 fluorescence was detected by PMT2 (Leica SP5) in the range of 530-590 nm and excited using the 476 nm line of an argon ion laser. Anthocyanins were detected as described before. Both fluorophores were recorded sequentially, line by line.

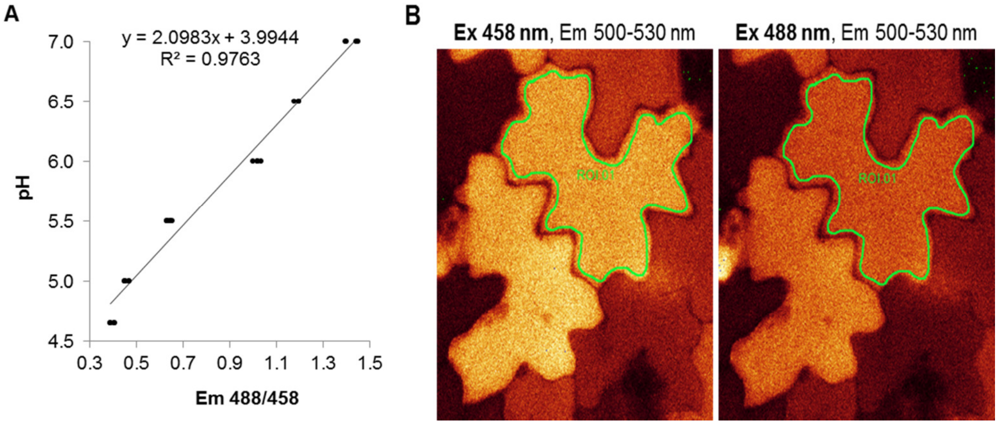

***In vivo* pH measurements**: (A) 6-CF calibration curve showing the ratio of emissions intensities, which were detected after excitation at 458 nm and 488 nm. pH values in vacuoles of epidermal cells were calculated as pH = $((Em_{488nm}/Em_{458nm}) * 2.0983) + 3.9944$. (B) Representative images of 6-CF fluorescence in epidermal cells after excitation at 458 nm (left image) and 488 nm (right image). Regions of interest (ROI, shown in green) were marked to calculate pH values for single cells.

Transmission electron microscopy

Samples were fixed for 1 hour by the addition of glutaraldehyde to the cell culture to give a 2.5% final concentration then pre-embedded in low-gelling temperature agarose before further fixation in 2.5% (v/v) glutaraldehyde in 0.05 M sodium cacodylate, pH 7.3, overnight. Samples were dehydrated through an ethanol series then gradually infiltrated with LR White resin (London Resin Company, Reading, Berkshire) before polymerisation at 60°C. Samples were sectioned using a Leica UC6ultramicrotome (Leica, Milton Keynes) to give ultrathin sections of approximately 90nm. Sections were stained with 2% (w/v) uranyl acetate for 1hr and 1% (w/v) lead citrate for 1 minute, washed in distilled water and air dried. Grids were viewed in a FEI Tecnai 20 transmission electron microscope (FEI UK Ltd, Cambridge, UK) at 200 kV and imaged using an AMT XR60 digital camera (Deben, Bury St Edmunds, UK) to record TIF files.

Sequences of Gateway primers used for cloning

Primer name	Primer sequence
B1Ptf35hF	5' GGGGACAAGTTTGTACAAAAAAGCAGGCTGGACC ATGATGCTACTTACTGAGCTTG 3'
B2 Ptf35hR	5' GGGGACCACTTTGTACAAGAAAGCTGGGTCCT ATGGTACATAAACATCCAATTGTAA 3'
Ci3MaT1B1 F	5' GGGGACAAGTTTGTACAAAAAAGCAGGCTGGAT GGCTTCCAATTCCATTGTTA 3'
Ci3MaT1B2 R	5' GGGGACCACTTTGTACAAGAAAGCTGGGTCT TATATCTCACTCTCTAATCCG 3'
B1AAT	5' GGGGACAAGTTTGTACAAAAAAGCAGGCTGG ATGGTGG CTCATCTTCAACCT 3'
B2AAT	5' GGGGACCACTTTGTACAAGAAAGCTGGGTCCGTTGCGAAT TTCTTGATCCC 3'
B1SAT	5' GGGGACAAGTTTGTACAAAAAAGCAGGCTGGATGAGCC AAATTACAACACAAAA 3'
B2SAT	5' GGGGACCACTTTGTACAAGAAAGCTGGGTCCTTTGGCACAT AACTAAACTC 3'
AtGF	5' GGGGACAAGTTTGTACAAAAAAGCAGGCTGGAT GGCCACTTCCGTCAATGG 3'
AtGR	5' GGGGACCACTTTGTACAAGAAAGCTGGGTCCTAC TCATCCTCGTCCACAAA 3'

Supplemental References

S1. Tohge, T., Nishiyama, Y., Hirai, M.Y., Yano, M., Nakajima, J.-i., Awazuhara, M., Inoue, E., Takahashi, H., Goodenowe, D.B., Kitayama, M., et al. (2005). Functional genomics by integrated analysis of metabolome and transcriptome of Arabidopsis plants over-expressing an MYB transcription factor. The Plant Journal *42*, 218-235.

S2. Luo, J., Nishiyama, Y., Fuell, C., Taguchi, G., Elliott, K., Hill, L., Tanaka, Y., Kitayama, M., Yamazaki, M., Bailey, P., et al. (2007). Convergent evolution in the BAHD family of acyl transferases: identification and characterization of anthocyanin acyl transferases from Arabidopsis thaliana. The Plant Journal *50*, 678-695.

S3. Tohge, T., Zhang, Y., Peterek, S., Matros, A., Rallapalli, G., Tandrón, Y.A., Butelli, E., Kallam, K., Hertkorn, N., Mock, H.-P., et al. (2015). Ectopic expression of snapdragon transcription factors facilitates the identification of genes encoding enzymes of anthocyanin decoration in tomato. The Plant Journal *83*, 686-704.

S4. Butelli, E., Titta, L., Giorgio, M., Mock, H.P., Matros, A., Peterek, S., Schijlen, E.G., Hall, R.D., Bovy, A.G., Luo, J., et al. (2008). Enrichment of tomato fruit with health-promoting anthocyanins by expression of select transcription factors. Nature Biotechnology *26*, 1301-1308.

S5. Holton, T.A., Brugliera, F., Lester, D.R., Tanaka, Y., Hyland, C.D., Menting, J.G.T., Lu, C.-Y., Farcy, E., Stevenson, T.W., and Cornish, E.C. (1993). Cloning and expression of cytochrome P450 genes controlling flower colour. Nature *366*, 276-279.

S6. Suzuki, H., Nakayama, T., Yamaguchi, M.A., and Nishino, T. (2004). cDNA cloning and characterization of two Dendranthema x morifolium anthocyanin malonyltransferases with different functional activities. Plant Science *166*, 89-96.

S7. Rodriguez-Saona, L.E., and Wrolstad, R.E. (2001). Extraction, Isolation, and Purification of Anthocyanins. In Current Protocols in Food Analytical Chemistry. (John Wiley & Sons, Inc.).

S8. Giusti, M.M., and Wrolstad, R.E. (2001). Characterization and Measurement of Anthocyanins by UV-Visible Spectroscopy. In Current Protocols in Food Analytical Chemistry. (John Wiley & Sons, Inc.).

S9. Seidel, T., Golldack, D., and Dietz, K.-J. (2005). Mapping of C-termini of V-ATPase subunits by in vivo-FRET measurements. FEBS Letters *579*, 4374-4382.
