# Supplementary material for: Aromatic Decoration Determines the Formation of Anthocyanic Vacuolar Inclusions
Source: Curr Biol. 2017 Apr 3;27(7):945–57. doi: 10.1016/j.cub.2017.02.027 (PMC5387179; doi:10.1016/j.cub.2017.02.027)
Supplement: Document S2. Article plus Supplemental Information [file mmc2.pdf]

Current Biology

Article

# Aromatic Decoration Determines the Formation of Anthocyanic Vacuolar Inclusions

## Highlights

- Anthocyaninic vacuolar inclusions (AVIs) are precipitates of acylated anthocyanins
- AVIs are formed only by aromatically acylated anthocyanins
- No other compounds are present in AVIs as shown by their reconstitution in vitro
- Glycosylation of acylated anthocyanins reduces their propensity to form AVIs

## Authors

Kalyani Kallam, Ingo Appelhagen, Jie Luo, ..., Øyvind M. Andersen, Kevin Davies, Cathie Martin

## Correspondence

cathie.martin@jic.ac.uk

## In Brief

Anthocyanins are red, purple, and blue pigments of flowers and fruits. They accumulate in vacuoles, and although usually soluble, in some plants they accumulate as structures termed anthocyanic vacuolar inclusions (AVIs). Kallam et al. show which decorations and conditions promote AVI formation and suggest a mechanism by which they form.

CrossMark

Kallam et al., 2017, Current Biology *27*, 945–957
April 3, 2017 © 2017 The Authors. Published by Elsevier Ltd.
http://dx.doi.org/10.1016/j.cub.2017.02.027

CellPress

Current Biology
Article

# Aromatic Decoration Determines the Formation of Anthocyanic Vacuolar Inclusions

Kalyani Kallam,[1] Ingo Appelhagen,[1] Jie Luo,[2] Nick Albert,[3] Huaibi Zhang,[3] Simon Deroles,[3] Lionel Hill,[1] Kim Findlay,[1] Øyvind M. Andersen,[4] Kevin Davies,[3] and Cathie Martin[1,5,*]
[1]Department of Metabolic Biology, John Innes Centre, Norwich NR4 7UH, UK
[2]National Key Laboratory of Crop Genetic Improvement and National Center of Plant Gene Research (Wuhan), Huazhong Agricultural University, Wuhan 430070, China
[3]New Zealand Institute for Plant and Food Research Limited, Private Bag 11-600, Palmerston North 4442, New Zealand
[4]Department of Chemistry, University of Bergen, Postboks 7803, 5020 Bergen, Norway
[5]Lead Contact
*Correspondence: cathie.martin@jic.ac.uk
http://dx.doi.org/10.1016/j.cub.2017.02.027

## SUMMARY

**Anthocyanins are some of the most widely occurring secondary metabolites in plants, responsible for the orange, red, purple, and blue colors of flowers and fruits and red colors of autumn leaves. These pigments accumulate in vacuoles, and their color is influenced by chemical decorations, vacuolar pH, the presence of copigments, and metal ions. Anthocyanins are usually soluble in the vacuole, but in some plants, they accumulate as discrete sub-vacuolar structures. Studies have distinguished intensely colored intra-vacuolar bodies observed in the cells of highly colored tissues, termed anthocyanic vacuolar inclusions (AVIs), from more globular, membrane-bound anthocyanoplasts. We describe a system in tobacco that adds additional decorations to the basic anthocyanin, cyanidin 3-*O*-rutinoside, normally formed by this species. Using this system, we have been able to establish which decorations underpin the formation of AVIs, the conditions promoting AVI formation, and, consequently, the mechanism by which they form.**

## INTRODUCTION

As early as 1905, Molisch observed that, in some plants, anthocyanin pigments accumulated as amorphous structures in contrast to their more usual, soluble form within the vacuoles of plant cells [1]. These membrane-bound bodies, enriched in anthocyanins, were suggested originally to be the sites of anthocyanin synthesis and called anthocyanoplasts. Anthocyanoplasts have been reported in over 70 species in 33 families of angiosperms [2]. Anthocyanins are synthesized in the cytoplasm but are actively transferred by a number of transporters across the tonoplast into the vacuole or into vesicles that then empty into the vacuole [3–5]. Anthocyanoplasts can fuse to form large anthocyanin-containing vesicles that may be cytoplasmic or vacuolar [6].

In contrast to anthocyanoplasts, anthocyanic vacuolar inclusions (AVIs) occur much less commonly. The term AVI was coined for non-membrane-bound vacuolar bodies composed of irregular thread-like structures in the petals of lisianthus (*Eustoma grandiflorum*) that are stable enough to purify [7, 8]. In lisianthus, fusion of prevacuolar compartments with the tonoplast and release of irregular thread-like structures (AVIs) have been observed in the darkly pigmented, inner epidermal cells at the base of the petals (Figure 1A), whereas distinct spherical bodies, which are probably anthocyanoplasts, can also be observed within the vacuole of more palely pigmented epidermal cells (Figure 1B) [7]. “Hard,” thread-like AVIs have also been purified from mutant sectors of dianthus [8] and grapevine [9, 10], but otherwise, localized concentrations of anthocyanins have proved recalcitrant to purification.

A clear distinction between non-membrane-bound, hard AVIs seen in the darkly colored, adaxial epidermal cells of lisianthus petals (Figure 1A) and membrane-bound, anthocyanin-rich bodies that predominate in cells of the lower abaxial petal epidermis of lisianthus (Figure 1B) was made by Grotewold and Davies [11]. Unfortunately, these authors then added to the confusion by subsequently referring to both as anthocyanic vacuolar inclusions. Here, we will adhere to the original definition of AVIs by Markham et al. [8] as non-membrane-bound, fibrous, vacuolar inclusions. On balance, it is likely that the formation of AVIs and the formation of membrane-bound, anthocyanin-rich vesicles involve different mechanisms.

Where AVIs have been purified, they have been shown to be composed of selected anthocyanins, possibly in association with specific proteins or lipids [8–10, 12]. Markham et al. [8] suggested that anthocyanins are sequestered in AVIs primarily to increase their stability and also to give more intense, bluer color. Formation of AVIs has been suggested to be dependent on acylation of anthocyanins, particularly coumaroylation (Figure 1C) [9, 10]. In some other species, the recruitment of anthocyanins into AVIs has been reported to be influenced strongly by the degree of glycosylation of the anthocyanins, as shown by compositional analysis of purified AVIs [8]. In most examples, high intracellular levels of anthocyanins are also closely associated with the formation of AVIs [9]. Different theories concerning the formation of AVIs have arisen from studies in widely different plants, where diverse factors could influence AVI formation. We developed a system to define the factors influencing AVI formation in a single plant species, tobacco, which does not normally produce AVIs.


This is an open access article under the CC BY license (http://creativecommons.org/licenses/by/4.0/).

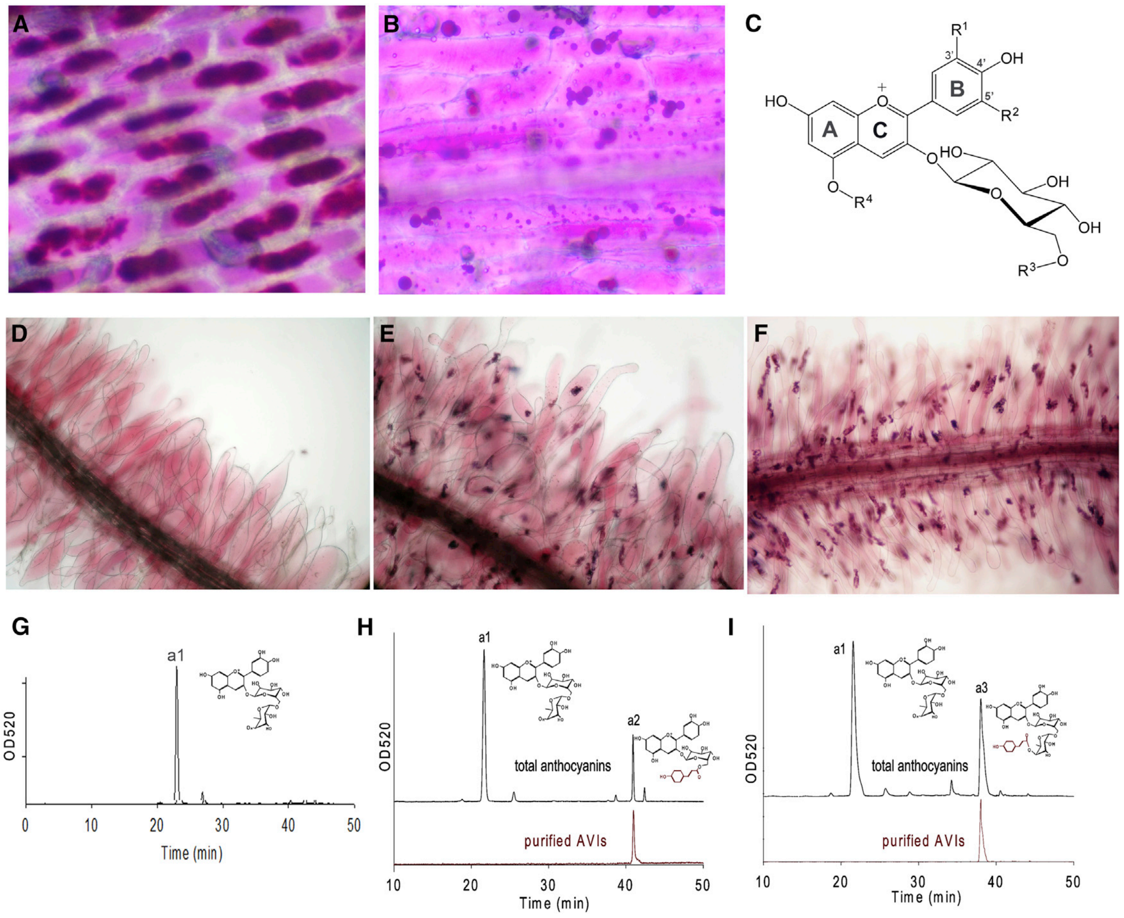


**Figure 1. Anthocyanin Accumulation and AVI Composition**

(A) AVIs in cells of the adaxial epidermis of the base of a lisianthus petal.

(B) Anthocyanoplasts or circular vesicles in cells of the abaxial epidermis of the mid part of a lisianthus petal.

(C) General structure of an anthocyanin molecule: $R^1$ = H, $R^2$ = H, $R^3$ = H, $R^4$ = H is pelargonidin 3-*O*-glucoside (Pel3G); $R^1$ = OH, $R^2$ = H, $R^3$ = H, $R^4$ = H is cyanidin 3-*O*-glucoside (Cy3G); $R^1$ = OH, $R^2$ = OH, $R^3$ = H, $R^4$ = H is delphinidin 3-*O*-glucoside (Del3G); $R^1$ = OH, $R^2$ = H, $R^3$ = rhamnosyl, $R^4$ = H is cyanidin 3-*O*-rutinoside (Cy3R); $R^1$ = OH, $R^2$ = OH, $R^3$ = rhamnosyl, $R^4$ = H is delphinidin 3-*O*-rutinoside (Del3R); $R^1$ = OH, $R^2$ = H, $R^3$ = coumaroyl, $R^4$ = H is cyanidin 3-*O*-(6″-*O*-(coumaroyl) glucoside) (Cy3couG); $R^1$ = OH, $R^2$ = OH, $R^3$ = coumaroyl, $R^4$ = H is delphinidin 3-*O*-(6″-*O*-(coumaroyl)glucoside) (De3couG); $R^1$ = OH, $R^2$ = H, $R^3$ = malonyl, $R^4$ = H is cyanidin 3-*O*-(6″-*O*-(malonyl) glucoside) (Cy3malG); $R^1$ = OH, $R^2$ = H, $R^3$ = H, $R^4$ = glucosyl is cyanidin 3,5-*O*-diglucoside (Cy3G5G); and $R^1$ = OH, $R^2$ = H, $R^3$ = coumaroyl, $R^4$ = glucosyl is cyanidin 3-*O*- (6″-*O*-(coumaroyl)glucoside)-5-*O*-glucoside (Cy3couG,5G).

(D) Root hairs of *Del/Ros1* tobacco seedling showing accumulation of high levels of soluble anthocyanins.

(E) Root hairs of *Del/Ros/At3AT* tobacco seedling showing the accumulation of soluble anthocyanins and anthocyanic vacuolar inclusions (AVIs) as dense, pigmented aggregates.

(F) Root hairs of *Del/Ros/Sl3AT* tobacco seedling showing AVIs as dense, pigmented aggregates.

(G) HPLC profile of total anthocyanin (cyanidin 3-*O*-rutinoside) of *Del/Ros1* lines.

(H) HPLC profiles of total anthocyanins (black, above) and anthocyanins extracted from purified AVIs (red, below) of *Del/Ros/At3AT* lines. Cyanidin 3-*O*-(6″-*O*-(coumaroyl) glucoside) was found in AVIs.

(I) HPLC profiles of total anthocyanins (black, above) and anthocyanins extracted from AVIs (red, below) of *Del/Ros/Sl3AT* lines. Cyanidin 3-*O*-(6″-*O*-(coumaroyl) rutinoside) was detected in AVIs.

See also Figure S1.

## RESULTS

### Aromatic Acylation of Anthocyanins Is Required for AVI Formation In Vivo

We first developed lines of tobacco that produced high levels of anthocyanins in every cell by constitutive expression of two transcription factors, *Delila* (*Del*) and *Rosea1* (*Ros1*) from snapdragon (*Antirrhinum majus*) [13, 14] (Figures 1D and 2Ai). Lines of *Del/Ros1* tobacco accumulated very high levels of cyanidin 3-*O*-rutinoside (Figures 1C and S1A; Table S1), exclusively in a soluble form within vacuoles (Figures 1D and 2Ai), and no AVIs were ever observed in tissues accumulating only cyanidin 3-*O*-rutinoside. However, when these lines were crossed to a line of tobacco constitutively expressing a cyanidin 3-*O*-glucoside, *p*-coumaroyl coenzyme A (CoA) acyltransferase from *Arabidopsis* (At3AT) (Table S1) [15], the progeny accumulated

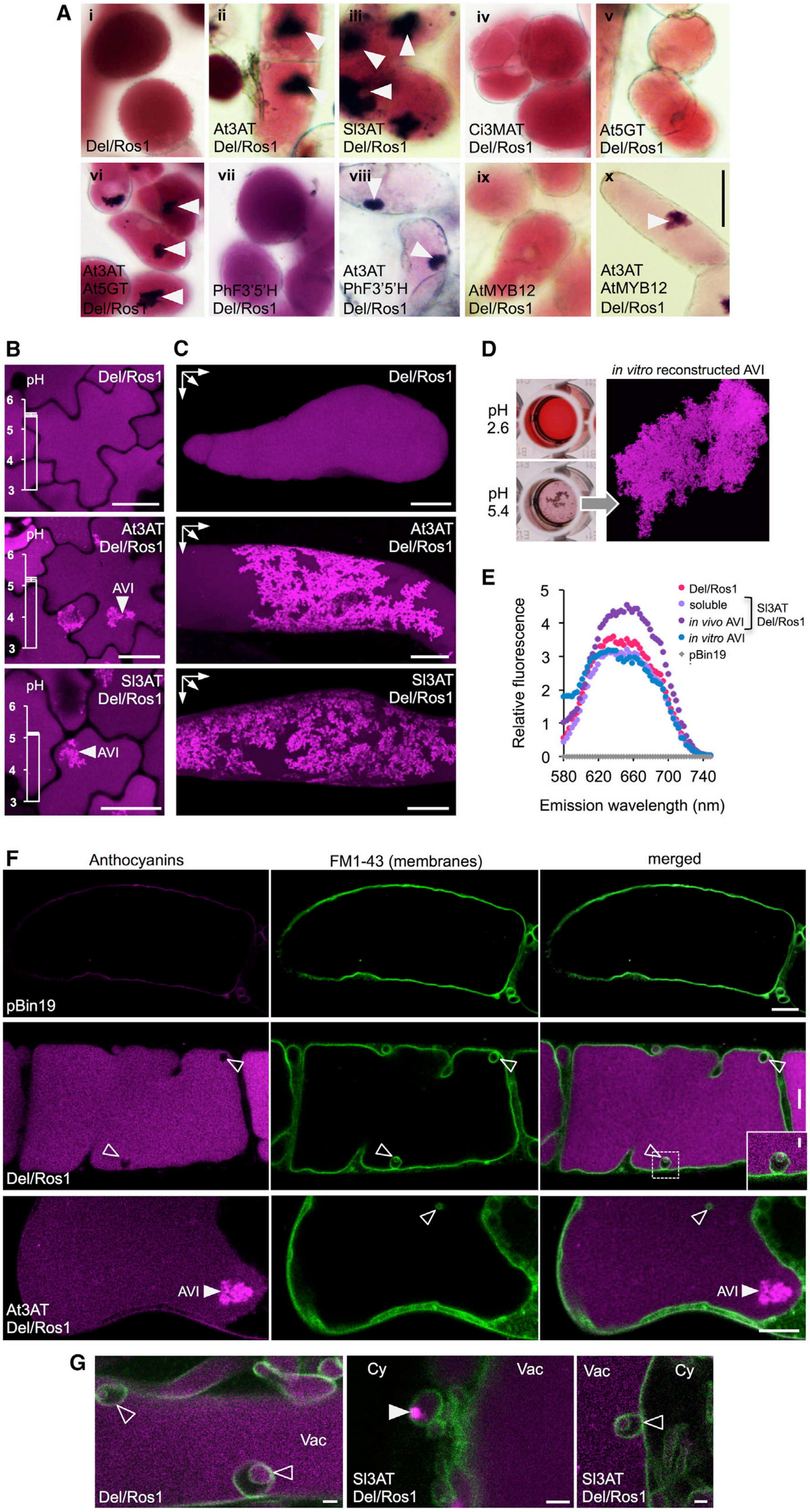


(legend on next page)

anthocyanins that were present in AVIs as well as soluble in the vacuole (Figures 1E and 2Aii). Analysis of the total anthocyanins in the *Del/Ros1/At3AT* plants revealed a mixture of cyanidin 3-*O*-rutinoside and smaller amounts of cyanidin 3-*O*-(6″-*O*-(coumaroyl)glucoside) (Figures 1H and S1B). However, purified AVIs from these plants contained only cyanidin 3-*O*-(6″-*O*-(coumaroyl)glucoside) (Figure 1H), suggesting that acylation might be required for anthocyanin accumulation in AVIs in tobacco.

Acylation of cyanidin 3-*O*-glucoside by At3AT prevents the addition of the rhamnosyl group found in cyanidin 3-*O*-rutinoside (Figure 1C), and so, theoretically, AVIs might form because of loss of the rhamnosyl group from cyanidin 3-*O*-rutinoside, as previously suggested for AVIs in lisianthus [8] rather than because of the presence of the aromatic acyl group in cyanidin 3-*O*-(6″-*O*-(*p*-coumaroyl)glucoside) [10]. Consequently, we expressed constitutively a second anthocyanin acyl transferase from tomato that adds a *p*-coumaroyl group to the 4‴ position of the rhamnose in cyanidin 3-*O*-rutinoside [16]. When lines constitutively producing this enzyme (Sl3AT) were crossed to *Del/Ros1* lines, the progeny also accumulated anthocyanins in both AVIs and in soluble form (Figures 1F and 2Aiii). Purified AVIs from these plants contained only cyanidin 3-*O*-(4‴-*O*-(*p*-coumaroyl)rutinoside), although the lines also accumulated cyanidin 3-*O*-rutinoside (Figure 1I). These lines produced more AVIs than the lines expressing *At3AT* in the *Del/Ros1* background (Figures 1E and 1F), probably because of their relatively higher levels of acylated anthocyanins (Figures S1B and S1C; Table S1), because the Sl3AT enzyme, unlike At3AT, does not compete with the endogenous rhamnosyl transferase for its anthocyanin acceptor substrate.

To determine whether hydroxylation of the B ring of the anthocyanins influences AVI formation, we expressed the enzyme encoding flavonoid 3′5′ hydroxylase (from petunia; Table S1) [17] in tobacco. These lines were crossed to *Del/Ros1* lines, and consequently, high levels of delphinidin 3-*O*-rutinoside accumulated in all cells of the progeny (Figure S1G). However, no AVIs formed in the vacuoles of *Del/Ros1/F3′5′H* plants (Figure 2Avii). When we expressed *At3AT* in these lines, delphinidin 3-*O*-(6″-*O*-(coumaroyl)glucoside) together with delphinidin 3-*O*-rutinoside were produced (Figure S1H) and AVI formation was observed in the vacuole of each cell (Figure 2Aviii). This implied that AVI formation was associated with acylated anthocyanins and that the type of anthocyanidin did not influence formation in vivo, substantially.

To address whether aliphatic acylation had a similar effect to aromatic acylation of anthocyanins, we expressed cyanidin

**Figure 2. AVI Formation In Vivo in Tobacco Lines Accumulating High Levels of Anthocyanins**

(A) Bright-field images of callus cells generated from stable transgenic lines accumulating high levels of cyanidin 3-*O*-rutinoside together with different decorated anthocyanins. (i) Callus cells from *Del/Ros1* lines accumulating high levels of cyanidin 3-*O*-rutinoside, (ii) *Del/Ros1/At3AT* accumulating cyanidin 3-*O*-(6″-*O*-(coumaroyl)glucoside), (iii) *Del/Ros1/Sl3AT* accumulating cyanidin 3-*O*-(6″-*O*-(coumaroyl)rutinoside), (iv) *Del/Ros1/Ci3MAT* accumulating cyanidin 3-*O*-(6″-*O*-(malonyl)glucoside), (v) *Del/Ros1/At5GT* accumulating cyanidin 3-*O*-glucoside-5-*O*-glucoside, (vi) *Del/Ros1/At3AT/At5GT* accumulating cyanidin 3-*O*-(6″-*O*-(coumaroyl)glucoside)-5-*O*-glucoside, (vii) *Del/Ros1/PhF3′5′H* accumulating delphinidin 3-*O*-rutinoside, (viii) *Del/Ros1/At3AT/PhF3′5′H* accumulating delphinidin 3-*O*-(6″-*O*-(coumaroyl)glucoside), (ix) *Del/Ros1/AtMYB12* accumulating cyanidin 3-*O*-rutinoside,and rutin, and (x) *Del/Ros1/AtMYB12/At3AT* accumulating cyanidin 3-*O*-(6″-*O*-(coumaroyl)glucoside) and rutin are shown. All lines produced significant levels of cyanidin 3-*O*-rutinoside and lower levels of the modified anthocyanins, as shown in Figure S1. Lines accumulating cyanidin 3-*O*-(6″-*O*-(malonyl)glucoside) (Figure S1D), cyanidin 3-rutinoside-5-*O*-glucoside (Figure S1E), and delphinidin 3-*O*-rutinoside (Figure S1G) did not form AVIs. Lines accumulating cyanidin 3-*O*-(6″-*O*-(coumaroyl)glucoside) (Figure S1B), cyanidin 3-*O*-(4‴-*O*-(coumaroyl)rutinoside) (Figure S1C) cyanidin 3-*O*-(6″- *O*-(coumaroyl)glucoside)-5-*O*-glucoside along with cyanidin 3-*O*-(6″-*O*-(coumaroyl)glucoside) (Figure S1F), and delphinidin 3-*O*-(6″-*O*- (coumaroyl)glucoside) (Figure S1H) formed AVIs (white arrows). Calluses accumulating high levels of anthocyanins and flavonols did not form AVIs (ix) unless aromatically acylated anthocyanins were also present (x). AVIs formed as dense pigmented aggregates and did not appear to have a surrounding membrane.

(B) Optical sections showing anthocyanin auto-fluorescence in the leaf epidermis of selected transgenic tobacco lines that were used to generate the callus cells, shown in (A). Genotypes are as indicated. AVIs appeared as bright fluorescent signals (white arrows) surrounded by soluble anthocyanins with lower fluorescence intensities in the *Del/Ros1/At3AT* and *Del/Ros1/Sl3AT* lines. Cells of *Del/Ros1* plants contained only soluble anthocyanins with constant anthocyanin fluorescence intensities. Graphs on the left side of each image show the pH measured in vacuoles of each line.

(C) Maximum-projection images of anthocyanin auto-fluorescence from z stacks of callus cells, indicating the three-dimensional structures of the vacuoles. AVIs were extended throughout large parts of the vacuole and were observed only in cells of the *Del/Ros1/At3AT* and *Del/Ros1/Sl3AT* lines.

(D) In vitro AVI reconstruction from anthocyanin extracts of *Del/Ros1/Sl3AT* lines with cyanidin 3-*O*-(6″-*O*-(coumaroyl)rutinoside) in McIlvaine's buffer at different pH (left panel) and auto-fluorescence of an in vitro reconstructed AVI (right panel) under the same imaging conditions used for epidermal and callus cells, shown in (B).

(E) Emission spectrum of soluble anthocyanins in vacuoles of *Del/Ros1* (pink dots) and *Del/Ros1/Sl3AT* cells (light purple dots), AVIs in *Del/Ros1/Sl3AT* cells (dark purple dots), and in vitro reconstructed AVIs (blue dots) from cyanidin 3-*O*-(6″-*O*-(coumaroyl)rutinoside extracts. All lines showed similar emission spectra, with higher fluorescence intensities of AVIs in *Del/Ros1/Sl3AT* cells compared to soluble anthocyanins. A pBin19 empty vector line without anthocyanin accumulation showed no fluorescence under the same conditions (gray dots).

(F) Optical section of FM1-43-stained callus cells of different tobacco lines (left panel), anthocyanin auto-fluorescence in the same cells (middle panel), and merged images (right panel) showing membranes in green and anthocyanins in magenta pseudo-colors. No anthocyanins were apparent in cells of tobacco transformed with the empty vector (pBin19, upper row). Middle row shows a cell of the *Del/Ros1* tobacco line producing high levels of cyanidin 3-*O*-rutinoside. Small arrows show vesicles stained by FM1-43 that either contain or do not contain anthocyanins. The inset shows an enlarged image of the region marked by a dotted line. Bottom row shows a cell of the *Del/Ros1/At3AT* tobacco line producing AVIs. The large white arrow indicates the AVI, which is not surrounded by a membrane but free in the vacuole.

(G) Close-up images of FM1-43-stained anthocyanin-rich cells as shown in the right-hand panel in (F). Intra-vacuolar membrane-bound bodies without anthocyanins containing a membrane-bound anthocyanin-filled vesicle (open arrow) are common features of anthocyanin-rich tobacco cells, independent of whether they produce aromatically acylated or non-acylated anthocyanins (left and right panels). Additionally, small bright fluorescent AVIs were found outside the vacuole in membrane-bound anthocyanin-filled bodies in the cytoplasm but only in lines producing aromatically acylated anthocyanins (middle panel). Cy, cytoplasm; Vac, vacuole.

Scale bars represent 30 μm in (A), 25 μm in (B) and (C), 10 μm in (F), and 2 μm in (G) and inset in (F). See also Figures S1, S2, and S5.

3-*O*-glucoside, malonyl transferase from *Chrysanthemum indicum* (*Ci3MaT1*) (Table S1) in tobacco [18]. This enzyme adds an aliphatic acyl group to the 6″ position of the anthocyanidin 3-*O*-glucoside to produce cyanidin 3-*O*-(6″-*O*-(malonyl)glucoside). Ci3MaT1 activity is equivalent to the activity of At3AT, except that a malonyl group is added instead of a coumaroyl group to the same position on the anthocyanin acyl acceptor (Figure S1D). Tobacco lines accumulating cyanidin 3-*O*-(6″-*O*-(malonyl)glucoside) in a high-anthocyanin environment (*Del/Ros1/ Ci3MaT1*) did not form AVIs (Figure 2Aiv). Our results showed that AVIs are formed in tobacco only by aromatically acylated anthocyanins when combined with high levels of anthocyanin accumulation.

Although we had shown that the formation of AVIs is dependent on anthocyanin acylation, glycosylation has been reported to affect the type or degree of AVI formation in lisianthus [8]. To understand the influence of 5-*O*-glucosylation on AVI formation in a high anthocyanin environment, cyanidin 3-*O*-glucoside-5-*O*-glucoside was engineered in tobacco by expressing *At5GT*, an enzyme encoding anthocyanin 5-*O*-glucosyl transferase from *Arabidopsis* [19] (Table S1; Figure S1E). AVIs were not observed in the cells of progeny plants (Figure 2Av).

To understand better the effect of 5-*O*-glycosylation on AVI formation by coumaroylated anthocyanins in tobacco, we generated tobacco lines expressing both *At3AT* and *At5GT* together with *Del* and *Ros1* for high-level anthocyanin production. *Del/Ros1/At3AT/At5GT* seedlings producing both cyanidin 3-*O*-(6″-*O*-(coumaroyl) glucoside)-5-*O*-glucoside and cyanidin 3-*O*-(6″-*O*-(coumaroyl)glucoside) (Table S1; Figure S1F) showed AVI formation in the vacuoles of the cells (Figure 2Avi).

To investigate whether flavonols could interfere with AVI formation, we generated tobacco lines expressing the AtMyb12 transcription factor, which induces flavonol production [20, 21], together with *Del/Ros1.* These lines showed no AVI formation (Figure 2Aix) whereas, when *At3AT* was expressed together with *AtMYB12* in *Del/Ros1* lines, formation of AVIs was observed (Figure 2Ax).

## AVIs in Tobacco Are Not Membrane Bound

Recently, Chanoca et al. [22] reported AVIs to be formed in *Arabidopsis* and lisianthus by a mechanism related to micro-autophagy. They suggested that anthocyanin aggregates form close to the tonoplast and are engulfed by a double membrane before being expelled into the vacuole. However, all the bodies enriched in anthocyanins described by these authors were membrane bound. The AVIs that form in the inner epidermis of lisianthus petals (Figure 1A) are not membrane bound [7, 8]. We examined sections of cultured tobacco cells producing high levels of anthocyanins by transmission electron microscopy (Figure S2). We observed membrane-bound vesicles similar to those reported by Chanoca et al. [22], associated with the tonoplast or free in the vacuole in tobacco cell lines producing high levels of different anthocyanins, but these were observed irrespective of whether the cells were producing non-acylated anthocyanins, aliphatically acylated anthocyanins, or aromatically acylated anthocyanins (Figures 2F and S2B–S2G). Staining of membranes coupled with anthocyanin fluorescence imaging indicated that the membrane-bound bodies, described by Chanoca et al. [22], are features of high-level anthocyanin production, possibly similar to prevacuolar compartments [7, 23, 24], rather than features necessarily associated with AVI formation (Figures 2F and 2G). Such membrane-bound bodies likely represent intermediate stages in anthocyanin transport to the vacuole and might be equivalent to "anthocyanoplasts". These vesicles were clearly distinct from the non-membrane-bound aggregates of anthocyanins observed in *Del/Ros1/At3AT* lines producing AVIs (Figure 2F). We occasionally observed anthocyanin-containing vesicles containing brighter fluorescent lumps of material (arrowed in middle panel of Figure 2G), suggesting AVIs can form in the vesicles on their way to the vacuole.

## Reconstitution of AVIs In Vitro

The properties of different anthocyanins are influenced by the presence of copigments, metal ions, and vacuolar pH [25, 26]. Having established the importance of aromatic acylation to AVI formation in vivo, we attempted to investigate the mechanism that triggers this process in vitro. Anthocyanins were purified from the leaves of the different tobacco lines that had been generated for this study (Figure S1; Table S1), including cyanidin 3-*O*-rutinoside, cyanidin 3-*O*-rutinoside-5-*O*-glucoside, cyanidin 3-*O*-(6″-*O*- (coumaroyl)glucoside), cyanidin 3-*O*-(6″-*O*-(malonyl)glucoside), cyanidin 3-*O*-(6″-*O*- (coumaroyl)glucoside)-5-*O*-glucoside, delphinidin 3-*O*-rutinoside, and delphinidin 3-*O*-(6″-*O*-(coumaroyl)glucoside) (Figure S3). We initially examined the behavior of the purified anthocyanins at 50 μM concentration in water. All were completely soluble. We then tested the effect of salt concentration, using both KCl and NaCl. At 37 mM NaCl, no precipitation of non-acylated anthocyanins was observed, whereas low levels of coumaroylated anthocyanins precipitated, and precipitation increased as the salt concentration increased (Figure 3A). The sensitivity of acylated anthocyanin precipitation to KCl concentration was lower, with clear precipitates forming only above 250 mM KCl (Figure 3A).

Precipitation of anthocyanins was also tested against pH using McIlvaine's buffer [27]. All anthocyanins were completely soluble at acidic pH (pH 2.6). However, solubility varied considerably above pH 4.5 or 4.6, depending on the type of side chain decoration (Figure 3B). Coumaroylated anthocyanins precipitated in McIlvaine's buffer (Figure 3B), 0.1 M sodium citrate buffer, and 0.4 M sodium acetate buffer, in and above pH 4.5. These precipitates resembled closely the AVIs observed in vivo (Figures 2A–2E). The precipitates formed by cyanidin 3-*O*-(6″-*O*-(coumaroyl) glucoside) in sodium acetate buffer at pH 4.5 and in McIlvaine's buffer at pH 7 were purified and analyzed by high-pressure liquid chromatography (HPLC), and the anthocyanins obtained from the in vitro precipitates were identical to each other and to the pure compounds (Figure S4), indicating that the precipitates were the same as the AVIs formed in vivo. Aliphatic acylation of anthocyanin (malonylation) in cyanidin 3-*O*-(6″-*O*-(malonyl)glucoside) did not lead to precipitation in vitro at any pH (Figure 3B).

Purified cyanidin 3-rutinoside-5-*O*-glucoside showed no precipitation at higher pH compared to coumaroylated anthocyanins, confirming our in vivo observations that 5-glucosylation does not promote AVI formation (Figure 3B). On the contrary, the presence of a 5-*O*-glucoside in cyanidin 3-*O*-(6″-*O*-(coumaroyl)glucoside)-5-*O*-glucoside resulted in substantially

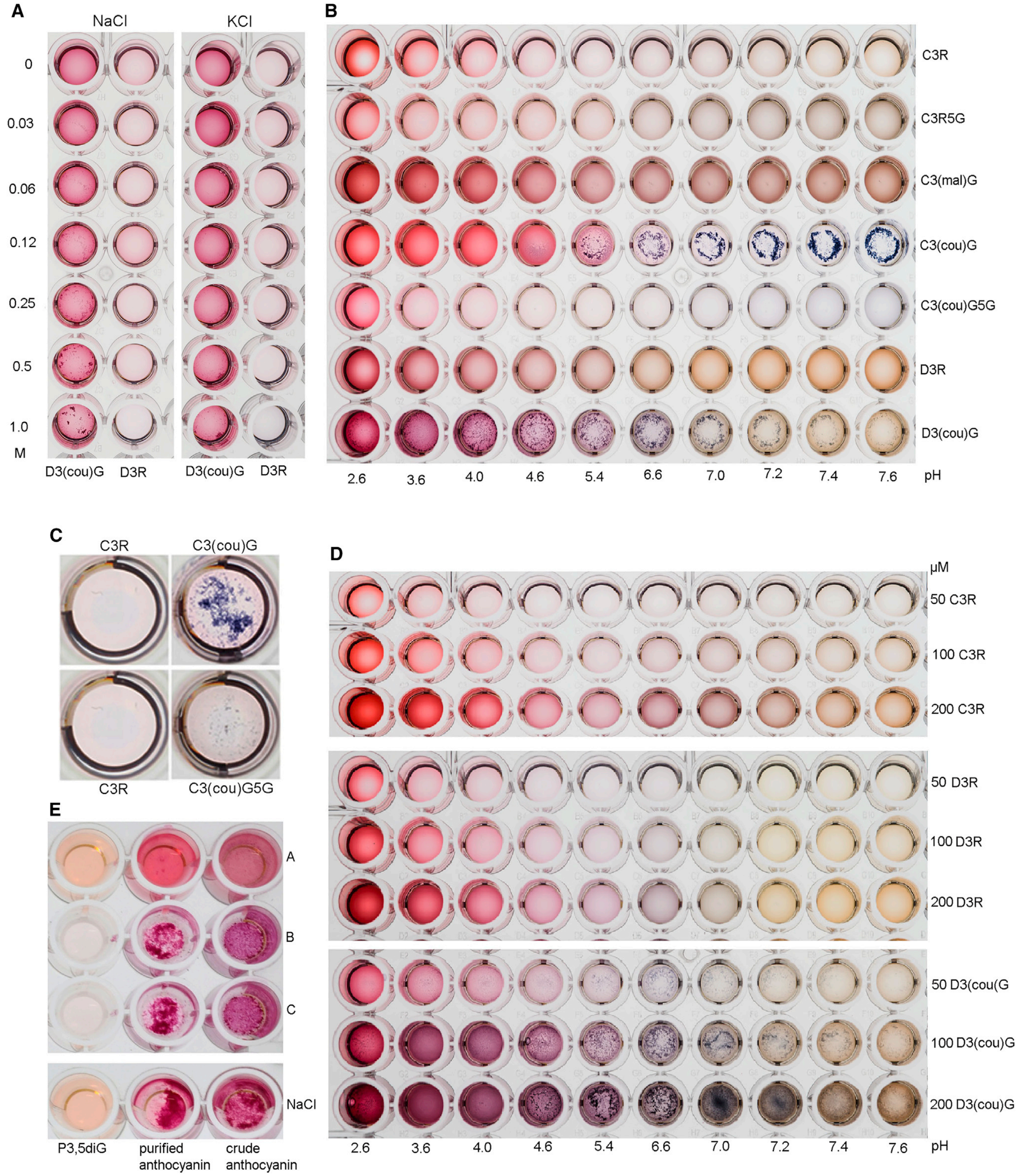


**Figure 3. Effects of Salt, pH, and Concentration on Precipitation of Anthocyanins In Vitro**

(A) The effects of increasing concentrations of salt were tested using KCl and NaCl. Delphinidin 3-*O*-(6″-*O*-(coumaroyl)glucoside) at 50 μM was tested.

(B) The effects of increasing pH on purified anthocyanins was studied in McIlvaine's buffer at increasing pH: 50 μM (C3R; cyanidin 3-*O*-rutinoside, C3R5G; cyanidin 3-*O*-rutinoside-5-*O*-glucoside), C3couG; cyanidin 3-*O*-(6″-*O*-(coumaroyl)glucoside); C3malG; cyanidin 3-*O*-(6″-*O*-(malonyl)glucoside), C3couG5G; cyanidin 3-*O*-(6″- *O*-(coumaroyl) glucoside)-5-*O*-glucoside, D3R; delphinidin 3-*O*-rutinoside), and D3couG; delphinidin 3-*O*-(6″-*O*- (coumaroyl)glucoside). AVI formation was clearly associated with coumaroylation and increased with increasing pH.

*(legend continued on next page)*

reduced precipitation in vitro at pH 4.5 compared to cyanidin 3-*O*-(6″-*O*- (coumaroyl)glucoside) in vitro (Figure 3C). Precipitates of anthocyanins from *Del*/*Ros1*/*At3AT*/*At5GT* line in 0.4 M sodium acetate buffer at pH 4.5, consisting of a mixture of cyanidin 3-*O*-rutinoside-5-*O*-glucoside, cyanidin 3-*O*-(6″-*O*-(coumaroyl)glucoside)-5-*O*-glucoside, cyanidin 3-*O*-(6″-*O*-(coumaroyl)glucoside), and cyanidin 3-*O*-rutinoside, were purified. They contained cyanidin 3-*O*-(6″-*O*-(coumaroyl)glucoside)-5-*O*-glucoside as well as cyanidin 3-*O*-(6″-*O*-(coumaroyl)glucoside) (Figure S4D), confirming that 5-*O*-glucosylation of anthocyanins does not completely preclude precipitation.

We tested the effects of increasing concentrations of anthocyanins on precipitation at different pH values. Precipitation in vitro was, unsurprisingly, greater with increasing concentrations of aromatically acylated anthocyanins (compare 200 μM to 50 μM in Figure 3D). This likely explains why, in planta, AVIs are associated with high levels of anthocyanin production. Aromatically acylated anthocyanins may comprise only a small proportion of the total anthocyanins in a vacuole, requiring high levels of overall production for AVIs to be clearly visible. Consequently, we never observed AVIs in the pale pink flowers of primary tobacco transformants expressing *At3AT* or *Sl3AT* alone. AVIs were observed in tobacco only when anthocyanin levels were enhanced by expression of *Del* and *Ros1* together with *At3AT* or *Sl3AT*. Interestingly, precipitation was seen with delphinidin 3-*O*-(6″-*O*-(coumaroyl)glucoside) even at 50 μM concentration and precipitation increased from pH 4.6 to 7.0 but decreased thereafter as the pH increased. At the higher concentrations of 100 and 200 μM of delphinidin 3-*O*-(6″-*O*-(coumaroyl)glucoside), AVI-like structures were formed between pH 4.0 to 6.6, equivalent to the vacuolar pH values we measured in the different tobacco lines and equivalent to pH values widely observed in fruits and flowers (Figures 2B and 3B). For cyanidin 3-*O*-(6″-*O*-(coumaroyl)glucoside), precipitation did not decrease above pH 7.0 (Figure 3B), supporting the suggestion that AVI formation is greater for cyanidin- than delphinidin-type anthocyanins [8] (at least at higher pH).

We also tested the effects of increasing concentrations of flavonols (quercetin -*O*-rutinoside; rutin) on precipitation of cyanidin 3-*O*-(6″-*O*-(coumaroyl)glucoside) in 0.1 M sodium citrate buffer (pH 4.5). No effect of rutin (0–100 mM) was observed on precipitation in vitro (Figure S5), confirming our in vivo observations (Figure 2Ax).

We compared the fluorescence spectra of AVIs formed in vivo in *Del*/*Ros1*/*Sl3AT* tobacco lines to those formed in vitro from anthocyanins extracted from these lines. These spectra were identical (Figures 2D and 2E), confirming the association between AVIs formed in vivo and the precipitates formed in vitro from aromatically acylated anthocyanins.

### Reconstitution of AVIs In Vitro from Lisianthus Anthocyanins

In the dark, central region of the inner epidermis of lisianthus petals where AVIs form (Figure 1A), the anthocyanins are aromatically acylated on the 5-*O*-glucoside, whereas the 3-*O*-glycoside residue is an undecorated galactoside. We extracted total anthocyanins from the dark basal regions of lisianthus petals in 80% acidified methanol. Dilution of these extracts to 50 μM anthocyanin in water (~pH 6.8) had no effect on anthocyanin solubility. Dilution of the anthocyanin extract from lisianthus to 50 μM in McIlvaine's buffer (pH 4.5) or 0.1 M sodium acetate buffer at pH 4.5 or 150 mM NaCl resulted in precipitation of anthocyanins (Figure 3E), similar to the response we had observed for the aromatically acylated anthocyanins from tobacco.

Although previous reports have suggested involvement of other compounds in AVI formation, including proteins and lipids [8, 12, 23, 28, 29], our in vitro studies suggested strongly that neither lipids nor proteins are essential for the formation of AVIs.

### AVI Formation In Vivo Is Dependent on pH

Anthocyanins themselves are good pH indicators. We measured the vacuolar pH of AVI- and non-AVI-producing tobacco cells, and the similarity in color of the soluble anthocyanins in AVI- and non-AVI-producing root hairs suggested that there were no substantial changes in vacuolar pH in AVI-producing cells (compare Figure 1D with Figures 1E and 1F). This was confirmed by measuring vacuolar pH in cells using the pH-dependent fluorescence of the ratiometric dye 6-carboxyfluorescein [30], and pH values between 5.2 and 5.5 were measured (Figure 2B), values which were very similar to cells that do not accumulate anthocyanins [31].

To confirm that AVI formation in vivo is dependent on pH, as observed for the precipitation of coumaroylated anthocyanins in vitro, we grew *Del*/*Ros1*/*At3AT* and *Del*/*Ros1*/*Sl3AT* seedlings on Murashige and Skoog (MS) medium with reduced nitrate. Nitrate was removed from the medium, and nitrogen was provided in the form of 0.2 mM ammonium chloride. Nitrate depletion of this type leads to acidification of plant cells [32, 33]. Seedlings germinated on this medium but became chlorotic after about 3 weeks. Root hairs of *Del*/*Ros1*/*At3AT* plants formed on nitrate-depleted medium, but they were shorter than the root hairs on regular MS medium and did not contain colored pigments or AVIs (Figures 4B1 and 4B2). The cells of the roots themselves retained color that was scarlet, indicating that the cells had acidified compared to the bluer-red color of roots and root hairs of *Del*/*Ros1*/*At3AT* plants grown on the regular MS medium

(C) 5-*O*-glucosylation increased anthocyanin solubility and reduced AVI formation in vitro, as evident from the reduced precipitation of 50 μM C3couG5G compared to 50 μM C3couG in sodium acetate buffer at pH 4.5.

(D) Effect of concentration of anthocyanin on AVI formation in vitro was studied in McIlvaine's buffer at increasing pH using purified anthocyanins: 50 μM; 100 μM; and 200 μM each (C3R; cyanidin 3-*O*-rutinoside, D3R; delphinidin 3-*O*-rutinoside, D3couG; delphinidin 3-*O*-(6″-*O*-(coumaroyl)glucoside)). The concentrations of anthocyanins tested were higher than in most natural situations. Irrespective of concentration, no AVI formation was seen with non-acylated anthocyanins in vitro.

(E) Pure pelargonidin 3-*O*-glucoside,5-*O*-glucoside (P3,5 diG), a crude extract of anthocyanins, and HPLC-purified anthocyanins from lisianthus were prepared at 50 μM in water (A), in sodium acetate buffer at pH 4.5 (B), in McIlvaine's buffer at pH 4.5 (C), and in 150 mM NaCl (NaCl). Both buffers at pH 4.5 as well as 150 mM NaCl gave rise to precipitation of anthocyanins (in vitro AVI formation).

See also Table S1 and Figures S3 and S4.

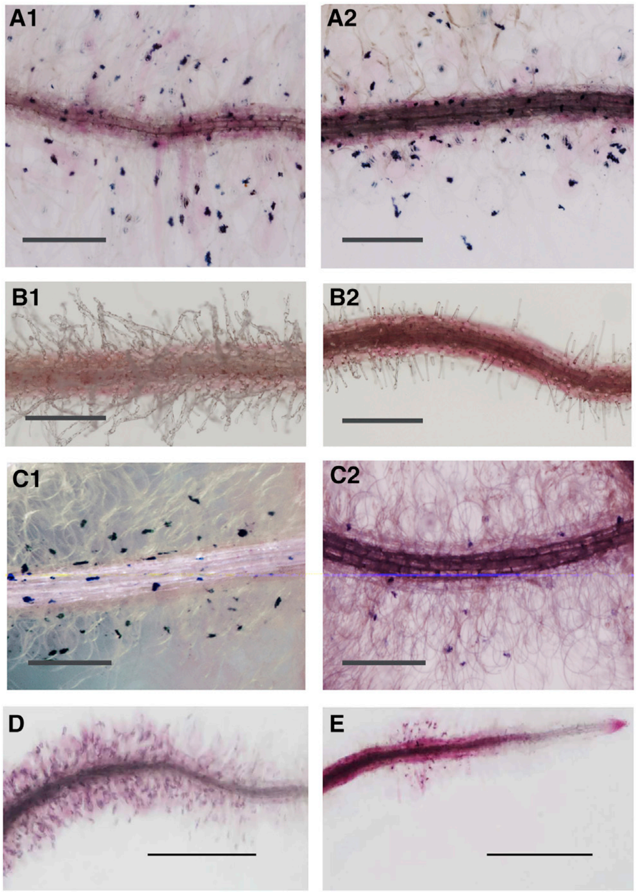


**Figure 4. Effect of Modification of pH on AVI Formation In Vivo**

(A1 and A2) Roots of *Del/Ros1/At3AT* seedlings grown on MS medium (0.6% agar).

(B1 and B2) Roots of *Del/Ros1/At3AT* seedlings grown on MS medium without nitrate but with 0.2 mM ammonium chloride (0.6% agar). Roots appeared redder, but no AVIs were seen in either the roots or root hairs.

(C1 and C2) Roots of *Del/Ros1/At3AT* seedlings returned to MS medium following growth on MS medium without nitrate but with 0.2 mM ammonium chloride (0.6% agar). AVIs were again apparent in root hairs and roots.

(D) Root of *Del/Ros1/Sl3AT* seedling grown on MS medium (0.6% agar).

(E) Root of *Del/Ros1/Sl3AT* seedling grown on MS medium without nitrate but with 0.2 mM ammonium chloride (0.6% agar). The color of the root is redder, and there are many fewer AVIs than seen on regular MS medium (D).

Scale bars represent 400 μm for (A), (B), and (C) and 1 mm for (D) and (E).

(Figures 4A1 and 4A2). Roots of seedlings grown on nitrate-depleted medium could be rescued by transfer back to regular MS medium, whereupon AVIs formed in their root hairs once again (Figures 4C1 and 4C2). *Del/Ros1/Sl3AT* plants developed more AVIs in their root hairs than *Del/Ros1/At3AT* plants, presumably due to the higher levels of coumaroylated anthocyanins in these lines (Figures S1 and 4D). When *Del/Ros1/Sl3AT* plants were grown on nitrate-depleted medium, the root hairs that developed had low levels of AVI production, and AVIs disappeared in the root hair cells as the roots grew into the acidifying medium (Figure 4E). These experiments demonstrated that AVI formation in vivo is dependent on pH, can be prevented by acidification of cells, and can be reinstated by re-alkalinization of cells.

## AVIs in Delphinium Flowers

Our studies with in vitro precipitates suggested that glucosylation of the 5-hydroxyl group might be critical in reducing the propensity of aromatically acylated anthocyanins to form AVIs. We searched for additional plants that might form AVIs by virtue of the fact that they made aromatically acylated anthocyanins lacking a 5-*O*-glycoside [34], and we selected two varieties of delphinium, *Morning Skies* and *King Arthur*. Delphinium *Morning Skies* produced pale blue flowers (Figure 5A), with AVIs that were clearly visible under bright-field illumination and using fluorescence imaging (Figure 5C). In contrast, delphinium *King Arthur* produced violet-colored sepals (Figure 5B), in which the anthocyanins were entirely soluble (Figure 5D). Staining of membranes and fluorescence imaging of sepal cells of *Morning Skies* showed that the AVIs in this variety were not membrane bound. However, numerous membrane-bound vesicles were observed within the vacuoles of these sepal cells, some containing anthocyanins, as indicated by their red fluorescence, and others not containing anthocyanins (Figure 5E).

Extraction of the anthocyanins from the sepals of *Morning Skies* showed these to be almost exclusively cyanodelphin (a tetra-p-hydroxybenzoylated anthocyanin; Figure 5H). Analysis of anthocyanins from sepals of *King Arthur* showed these to be almost exclusively violdelphin, which lacks three sugars and two hydroxybenzoyl groups compared to cyanodelphin (Figures 5H and 5I). We tested the extracted anthocyanins for their ability to make AVIs in vitro. Anthocyanins from *Morning Skies* were soluble at pH 2.6 (McIlvaine's buffer) but showed considerable precipitation when the pH increased to 3.6. This precipitation occurred up to pH 7.0 but appeared to decline compared to the precipitation at pH 3.6. No precipitation was observed at any pH for anthocyanins extracted from sepals of *King Arthur* at 6 $\mu g\ mL^{-1}$ nor at 30 $\mu g\ mL^{-1}$ (Figure 5F). The fluorescence of the in vitro AVIs from *Morning Skies* was identical to the fluorescence from its in vivo AVIs (Figures 5E and 5G).

## DISCUSSION

Anthocyanins exist in solution in several inter-convertible forms (secondary structures) with distinct colors (Figure 6): the flavylium cation; the hemiketals; the quinoidal forms; and the chalcone forms. The proportion of each anthocyanin adopting the various secondary structures is heavily influenced by pH, decoration, and other factors [26], and exact knowledge of this distribution is very limited for most anthocyanins under both in vitro and in vivo conditions. Figure 3B shows that the pH-dependent reactions in slightly acidic aqueous solutions convert flavylium cations to either colorless hemiketals or to purple/blue quinoidal forms, depending on the decoration of the anthocyanin. Unlike non-coumaroylated anthocyanins, both the 3-*O*-glucosides of delphinidin and cyanidin and to a lesser extent cyanidin 3-*O* (6″-*O*-(coumaroyl)glucoside)-5-*O*-glucoside precipitate in slightly acidic solutions when they are at comparable concentrations. The colors of these precipitates are in accordance with purple/blue quinoidal forms. We propose that the aromatic acyl group folds over the pyrylium ring (C-ring of the anthocyanidin) and protects the C-2 position from nucleophilic water attack, so favoring the existence of the colored forms.

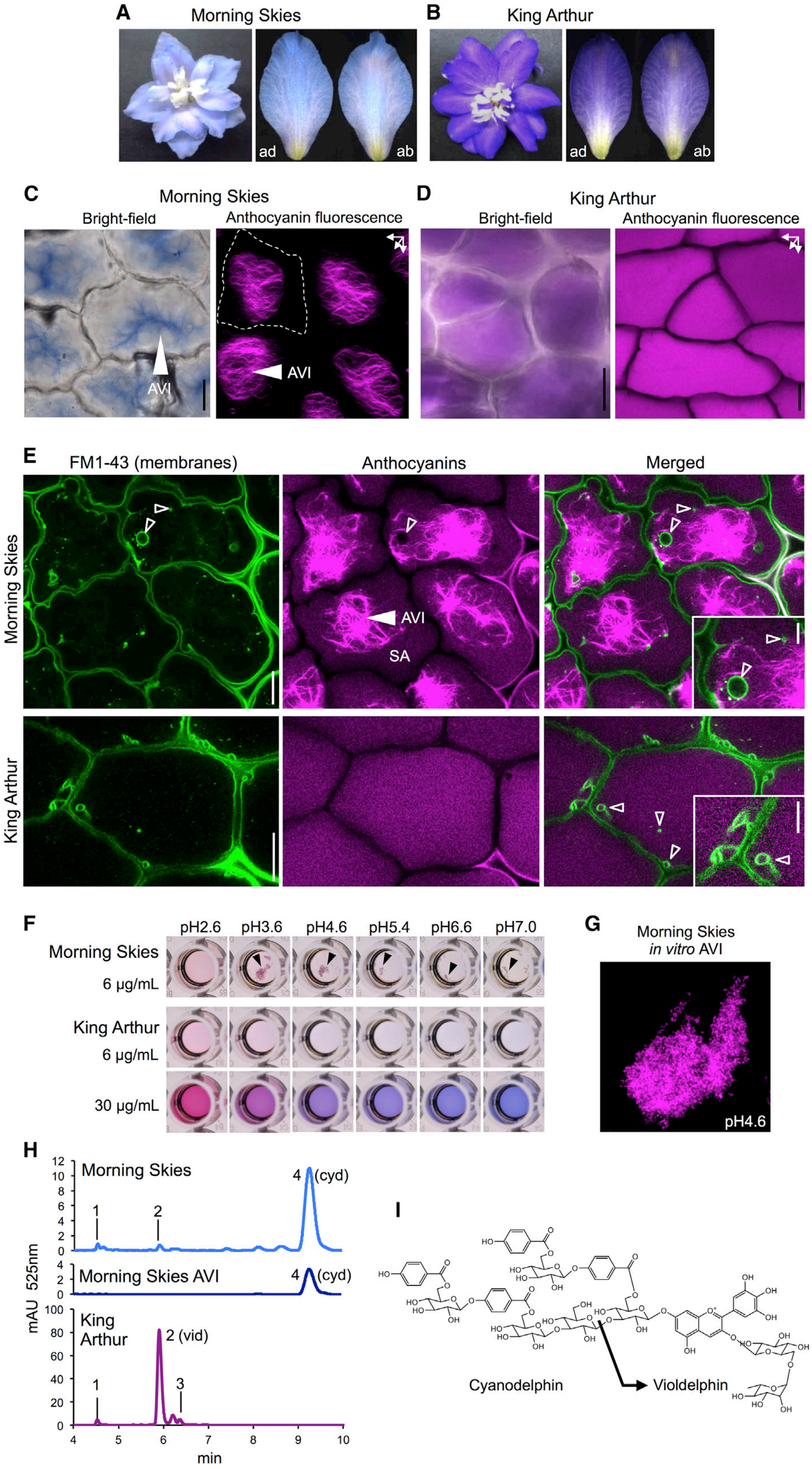


(legend on next page)

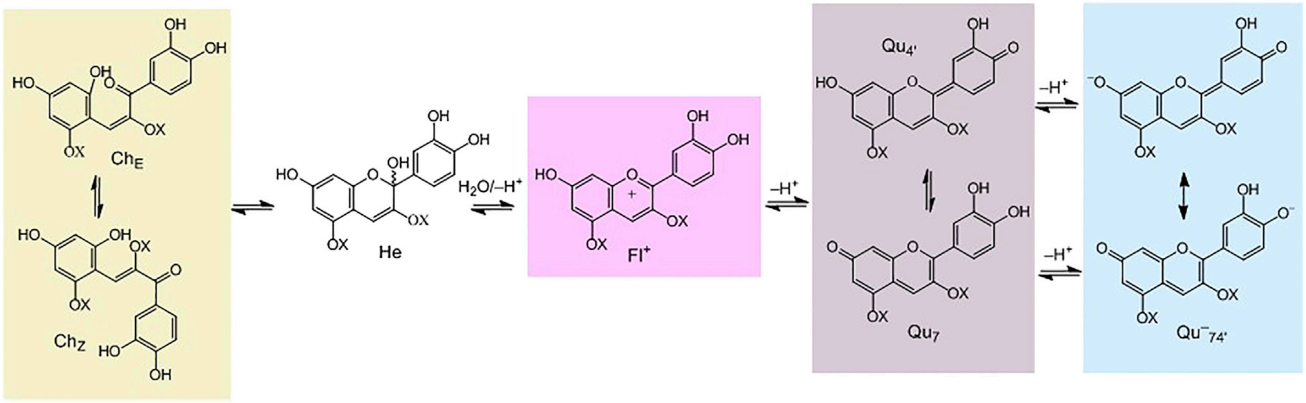


**Figure 6. Structural Transformations of Anthocyanidin Forms as a Function of pH**

$C_E$ and $C_Z$, chalcones (retrochalcones); Fl, flavylium cation; He, hemiketals (carbinol pseudobases); $Qu_7$ and $Qu_{4'}$, quinoidal bases; $Qu^-_{74'}$, ionized quinoidal base; X, glycosyl. The individual cyanidin forms are given with their colors as background.

The ensuing precipitation of anthocyanins in their quinoidal forms serves to maintain these colored forms over time. Without this precipitation, the remaining flavylium cations, in equilibrium with the quinoidal forms, would with time, shift toward the colorless, more thermodynamically stable hemiketals, as seen for the soluble anthocyanins without aromatic acyl groups in Figures 3B and 3C. Precipitation of anthocyanins was not observed for cyanidin 3-*O*-(6″-*O*-(malonyl)glucoside), an analogously structured anthocyanin acylated with an aliphatic acyl group.

This suggested mechanism for precipitation is supported by the effects of increasing salt concentration, which promote precipitation of the anthocyanins with aromatic acylation (Figure 3A). Salts will increase the ionic strength of the water surrounding the anthocyanidin and reduce the power of the water molecules as nucleophiles acting on the flavylium cation, thus reducing their probability of forming colorless hemiketals. Aromatic acyl groups are known to influence the secondary structures of anthocyanins. For instance, Fernandes et al. [35] compared the structure of malvidin 3-*O*-glucoside and malvidin 3-*O*-(6″-(coumaroyl) glucoside) as flavylium cations in acidic solutions by nuclear magnetic resonance (NMR). They concluded that acyl substituents seem to favor flavylium cation aggregation by two processes of interaction: formation of intramolecular complexes that involve both the double bond and the coumaric acid aromatic ring and intermolecular interactions resulting in the formation of larger aggregates [35].

The presence of a 5-*O*-glucoside in cyanidin 3-*O*-(6″-*O*-(coumaroyl)glucoside)-5-*O*-glucoside resulted in substantially reduced precipitation compared to cyanidin 3-*O*-(6″-*O*- (coumaroyl)glucoside) (Figure 3C). The extra glucosyl moiety at the 5-*O*-position of the anthocyanidin may explain the greater solubility of cyanidin 3-*O*-(6″-*O*-(coumaroyl)glucoside)-5-*O*-glucoside compared to cyanidin 3-*O*-(6″-*O*-(coumaroyl)glucoside). Alternatively, addition of glucose at the 5-hydroxyl position or elsewhere might reduce the influence of the aromatic acyl group in intramolecular stacking.

**Figure 5. AVI Formation in Delphinium Flowers**

(A) Open flowers of the light blue and bluish-purple delphinium cultivars *Morning Skies* (left) and *King Arthur* (right).

(B) Sepals of flowers as shown in (A). ab, abaxial side; ad, adaxial side.

(C) Bright-field image (left) and z stack maximum-projection image of anthocyanin auto-fluorescence (right) of adaxial sepal cells of delphinium *Morning Skies*. AVIs (white arrows) appeared as reddish to bluish amorphous structures in bright field. Soluble anthocyanins were barely visible. Fluorescence images were taken with low gain to avoid saturation of signals from AVIs. Soluble anthocyanins were not visible under these conditions due to their weaker fluorescence intensity. A single cell is outlined by a dotted line.

(D) Bright-field image (left) and maximum-projection image of anthocyanin auto-fluorescence (right) of adaxial sepal cells of delphinium *King Arthur* comparable to images for *Morning Skies*, shown in (C). Both images show only soluble anthocyanins and no amorphous structures nor differences in anthocyanin fluorescence intensities in contrast to *Morning Skies*.

(E) Optical section of FM1-43-stained adaxial sepal cells (left panel), anthocyanin auto-fluorescence in the same cells (middle panel), and merged images (right panel) showing membranes in green and anthocyanins in magenta pseudo-colors. Sepal cells of *Morning Skies* (upper row) contained soluble anthocyanins (SA) and brightly fluorescent AVIs in their vacuoles (filled arrow), as well as intra-vacuolar membrane-bound domains of different sizes (open arrows). AVIs were absent in *King Arthur* (lower panel) but contained intra-vacuolar membranes similar to *Morning Skies*.

(F) In vitro AVI reconstruction from anthocyanin crude extracts in McIlvaine's buffer at changing pH. Anthocyanin precipitates (black arrows) were most prominent in extracts from *Morning Skies* at pH 3.6 and 4.6 but were absent at pH 2.6 (upper panel). No precipitation is seen with anthocyanins from *King Arthur* flowers, at the same concentration as anthocyanins from *Morning Skies* (middle panel) or with five times higher concentration (lower panel).

(G) Auto-fluorescence of insoluble anthocyanins from *Morning Skies* in McIlvaine's buffer (pH 4.6).

(H) HPLC chromatogram of methanol extracts from *Morning Skies* flowers (light-blue line), in-vitro-reconstructed AVIs (dark blue line), and *King Arthur* flowers (purple line), recorded at 525 nm. Numbered peaks were identified by liquid chromatograph mass spectrometry-ion trap-time of flight. The main compounds are violdelphin (vid) (peak 2) and cyanodelphin (cyd) (peak 4).

(I) Structures of violdelphin and cyanodelphin. For peak identification, see Table S2.

Scale bars represent 1 mm in (B); 25 $\mu$m in (C), (D), and (E); and 5 $\mu$m in the insets in (E).

In nature, bluer shades in flowers have arisen, among other mechanisms, by the decoration of anthocyanins with aromatic acyl groups [26, 36]. The greater solubility of aromatically acylated anthocyanins following 5-*O*-glucosylation might provide an explanation for why aromatically acylated anthocyanins quite frequently have accompanying 5-*O*-glycosides, namely to maintain the solubility of the bluer, aromatically acylated anthocyanins within the solvent conditions prevalent in the vacuole of plant cells. A recent report suggested that 5-*O*-glucosylation of anthocyanins may have evolved early in angiosperms and been lost repeatedly in species lacking 5-*O*-glycosylated anthocyanins [16]. We have found 392 different anthocyanin structures with 5-*O*-glycosylation [37]. From this list, we removed 62 structures because they had not been characterized rigorously. Among the remaining 330 anthocyanins with 5-*O*-glycosylation, as many as 286 are acylated with either aliphatic and/or aromatic acyl groups. Of these, 135 have aliphatic acylation (mainly with malonic acid) of the sugar moiety located in the anthocyanidin 5-*O*-position. These structures are distributed in a variety of species within many families, and selection pressure for the retention of anthocyanin 5-*O*-glucosyl transferase (5GT) activity would allow the possibility to add an aliphatic acyl group, such as a malonyl group, to increase anthocyanin stability [15] or solubility. Interestingly, among the remaining 151 species with aromatically acylated anthocyanins, only genera *Eustoma* and *Gentiana* (both family Gentiaceae) and the unrelated species, *Browallia speciosa* [38], have been reported to contain anthocyanins with aromatic acylation of sugars located at the anthocyanidin 5-*O*-position. We conclude that attachment of aromatic acyl groups to the 5-*O*-glycoside has not been a prominent selective force for retaining 5-*O*-glycosylation, unlike malonylation. Perhaps it is the protective effect of 5-*O*-glucosylation against precipitation of aromatically acylated anthocyanins that means that 5GT activity has been retained in many dicot species with aromatically acylated anthocyanins.

The fact that aromatic acylation causes a bathochromic shift and a resultant bluer color by promoting intramolecular copigmentation or self-association involving quinoidal bases explains the bluing effect of AVI formation observed by Markham et al. [8] in blue-gray carnation and by others in other species [9]. The greater solubility of aromatically acylated anthocyanins with increasing glycosylation between pH 4.5 and 6.5 explains two additional observations of AVI formation made in different species. First, AVIs in purple lisianthus petals are enriched in the 3-galactoside, 5-(*E*-coumaroyl)glucoside and 3-galactoside, 5-(*E*-feruloyl)glucoside of delphinidin and cyanidin compared to the 3-(rhamnosyl)galactoside, 5-(*E*/*Z*-coumaroyl) glucoside and 3-(rhamnosyl)galactoside, 5-(*E*-feruloyl)glucoside of delphinidin in vacuolar solution [8]. This has been interpreted in terms of reduced glycosylation favoring AVI formation, which fits well with our in vitro observations, whereby 5-*O*-glycosylation reduced AVI formation in tobacco. Second, AVI formation in *Arabidopsis* is reportedly enhanced in the *5gt* mutation that knocks out the activity of the anthocyanidin 5-*O*-glucosyl transferase [22, 39]. *Arabidopsis* produces cyanidin 3-*O*-(6″-*O*-(4-*O*-(glucosyl)coumaroyl)-2′′-*O*-(2-*O*-(sinapoyl) xylosyl)glucoside)-5-*O*-(6-*O*-(malonyl)glucoside) as its major anthocyanin [40]. Removal of the 5GT activity and the accompanying stabilizing aliphatic acyl group from the anthocyanins in the *5gt* mutant might increase the relative proportion of the quinoidal forms and consequently the degree of precipitation of this anthocyanin in the vacuoles of cells producing high levels of anthocyanins.

This model for AVI formation is supported by our data from delphinium. Different varieties of delphinium producing cyanodelphin or violdelphin produced or did not produce AVIs (both in vivo and in vitro), respectively. The additional aromatic acyl groups in cyanodelphin likely have greater potential for association with the anthocyanidin than those in violdelphin, so protecting the C-2 position from hydration and the formation of the hemiketals. However, the promotion of quinoidal forms (and consequent AVI formation) is dependent on the number and type of decorating aromatic acyl groups, because violdelphin does not form AVIs whereas cyanodelphin does. This distinction is likely to be due to the orientation of the aromatic acyl groups in the anthocyanins, as determined by their own structures (for example, promotion of quinoidal forms may differ between *p*-coumaroyl and hydroxybenzoyl acyl groups), their attachments to different sugars, and their positions of attachment on the anthocyanidin molecule.

We suggest that AVIs form when the concentrations of aromatically acylated anthocyanins (or other anthocyanins with a high preponderance of quinoidal forms) reach a level that aggregates can form when the pH of the compartment is between 4.5 and 6.5. This probably occurs in the vesicles transporting the anthocyanins to the vacuole, which likely deliver both AVIs and soluble anthocyanins to the vacuole [7, 23] (Figure 2G). The requirements for relatively high levels of aromatically acylated anthocyanins and pH in the appropriate range for AVI formation will be met following active transport into the vesicles that convey both soluble anthocyanins and AVIs to the vacuole [5]. The formation of AVIs is most likely an unavoidable feature of the chemistry of some aromatically acylated anthocyanins, and it is possible that 5-*O*-glycosylation and additional glycosylation of the 3-*O*-glycoside have been retained or have evolved, respectively, to reduce any negative effects of large amounts of precipitates in the vesicles and the vacuole. However, in some instances, AVI formation may have been harnessed to enhance the intensity of pigmentation of plant tissues, as suggested in the case of the central black region of the petals of lisianthus [8].

## EXPERIMENTAL PROCEDURES

### Generation of Plant Material and Growth Conditions

Stable transgenic tobacco plants (*Nicotiana tabacum* ''Samsun'') were generated through *Agrobacterium tumefaciens* (LBA4404)-mediated transformation using binary constructs expressing all genes under the control of the double 35S promoter (for details, see Supplemental Experimental Procedures).

### Anthocyanin Extraction

Flower petals were extracted in 70% methanol with 0.1% HCl, 40 $\mu$L mg$^{-1}$, and leaf material was extracted using 0.01% HCl-acidified water (for details, see Supplemental Experimental Procedures).

### Preparative HPLC Purification

Anthocyanins were purified using a Gilson Preparative High Pressure Liquid Chromatography (Prep HPLC) System (for details, see Supplemental Experimental Procedures).

## SUPPLEMENTAL INFORMATION

Supplemental Information includes five figures, two tables, and Supplemental Experimental Procedures and can be found with this article online at http://dx.doi.org/10.1016/j.cub.2017.02.027.

## AUTHOR CONTRIBUTIONS

Investigation and methodology: K.K. and J.L. produced and characterized transgenic tobacco lines. K.K. purified the anthocyanins and studied AVI formation in vivo and anthocyanin precipitation in vitro under different conditions. I.A. performed confocal imaging of AVIs in tobacco and delphinium, and L.H. performed LC-MS runs and analysis. K.F. undertook transmission electron microscopy of tobacco cells. S.D. conducted analysis of lisianthus anthocyanins in vitro, N.A. contributed to identification of AVIs in tobacco, H.Z. conducted AVI isolation and contributed to data relating anthocyanin structure to AVI formation, and Ø.M.A. interpreted the relationships between structures of anthocyanins and their behaviors in solution. Conceptualization: S.D., H.Z., and K.D. contributed to experimental design and data interpretation for lisianthus. Writing the manuscript: K.K., K.D., Ø.M.A., I.A., and C.M. co-wrote the manuscript. All authors read and approved the manuscript.

## ACKNOWLEDGMENTS

We thank Baldeep Kular and Paul Needs for assistance with preparative HPLC; Andrew Davis for photography; and Tony Miller, Julia Russel, and Elaine Barclay for specialized technical input. K.K. and C.M. were supported by the European Union FP6 FLORA project (FOOD-CT-01730) and the European Union FP7 ATHENA collaborative project (grant agreement 245121), and I.A. and C.M. were supported by the ERA-NET Industrial Biotechnology project ANTHOPLUS (grant agreement BB/M004864/1). K.K. was supported by a Royal Society Dorothy Hodgkin Fellowship. C.M. is currently supported by the Institute Strategic Program *Understanding and Exploiting Plant and Microbial Secondary Metabolism* (BB/J004596/1) from the BBSRC.

Received: December 9, 2016
Revised: February 9, 2017
Accepted: February 13, 2017
Published: March 16, 2017

## REFERENCES

1. Molisch, H. (1905). Uber amorphes und krystallisiertes Anthokyan. Bot. Zeitung *63*, 145–162.
2. Pecket, R.C., and Small, C.J. (1980). Occurrence, location and development of anthocyanoplasts. Phytochemistry *19*, 2571–2576.
3. Goodman, C.D., Casati, P., and Walbot, V. (2004). A multidrug resistance-associated protein involved in anthocyanin transport in Zea mays. Plant Cell *16*, 1812–1826.
4. Francisco, R.M., Regalado, A., Ageorges, A., Burla, B.J., Bassin, B., Eisenach, C., Zarrouk, O., Vialet, S., Marlin, T., Chaves, M.M., et al. (2013). ABCC1, an ATP binding cassette protein from grape berry, transports anthocyanidin 3-O-Glucosides. Plant Cell *25*, 1840–1854.
5. Gomez, C., Terrier, N., Torregrosa, L., Vialet, S., Fournier-Level, A., Verriès, C., Souquet, J.M., Mazauric, J.P., Klein, M., Cheynier, V., and Ageorges, A. (2009). Grapevine MATE-type proteins act as vacuolar H+-dependent acylated anthocyanin transporters. Plant Physiol. *150*, 402–415.
6. Nozzolillo, C., and Ishikura, N. (1988). An investigation of the intracellular site of anthocyanoplasts using isolated protoplasts and vacuoles. Plant Cell Rep. *7*, 389–392.
7. Zhang, H., Wang, L., Deroles, S., Bennett, R., and Davies, K. (2006). New insight into the structures and formation of anthocyanic vacuolar inclusions in flower petals. BMC Plant Biol. *6*, 29.
8. Markham, K.R., Gould, K.S., Winefield, C.S., Mitchell, K.A., Bloor, S.J., and Boase, M.R. (2000). Anthocyanic vacuolar inclusions–their nature and significance in flower colouration. Phytochemistry *55*, 327–336.
9. Mizuno, H., Hirano, K., and Okamoto, G. (2006). Effect of anthocyanin composition in grape skin on anthocyanic vacuolar inclusion development and skin coloration. Vitis *45*, 173–177.
10. Conn, S., Zhang, W., and Franco, C. (2003). Anthocyanic vacuolar inclusions (AVIs) selectively bind acylated anthocyanins in *Vitis vinifera* L. (grapevine) suspension culture. Biotechnol. Lett. *25*, 835–839.
11. Grotewold, E., and Davies, K. (2008). Trafficking and sequestration of anthocyanins. Nat. Prod. Commun. *3*, 1251–1258.
12. Nozue, M., Yamada, K., Nakamura, T., Kubo, H., Kondo, M., and Nishimura, M. (1997). Expression of a vacuolar protein (VP24) in anthocyanin-producing cells of sweet potato in suspension culture. Plant Physiol. *115*, 1065–1072.
13. Goodrich, J., Carpenter, R., and Coen, E.S. (1992). A common gene regulates pigmentation pattern in diverse plant species. Cell *68*, 955–964.
14. Schwinn, K., Venail, J., Shang, Y., Mackay, S., Alm, V., Butelli, E., Oyama, R., Bailey, P., Davies, K., and Martin, C. (2006). A small family of MYB-regulatory genes controls floral pigmentation intensity and patterning in the genus Antirrhinum. Plant Cell *18*, 831–851.
15. Luo, J., Nishiyama, Y., Fuell, C., Taguchi, G., Elliott, K., Hill, L., Tanaka, Y., Kitayama, M., Yamazaki, M., Bailey, P., et al. (2007). Convergent evolution in the BAHD family of acyl transferases: identification and characterization of anthocyanin acyl transferases from Arabidopsis thaliana. Plant J. *50*, 678–695.
16. Tohge, T., Zhang, Y., Peterek, S., Matros, A., Rallapalli, G., Tandrón, Y.A., Butelli, E., Kallam, K., Hertkorn, N., Mock, H.-P., et al. (2015). Ectopic expression of snapdragon transcription factors facilitates the identification of genes encoding enzymes of anthocyanin decoration in tomato. Plant J. *83*, 686–704.
17. Holton, T.A., Brugliera, F., Lester, D.R., Tanaka, Y., Hyland, C.D., Menting, J.G.T., Lu, C.-Y., Farcy, E., Stevenson, T.W., and Cornish, E.C. (1993). Cloning and expression of cytochrome P450 genes controlling flower colour. Nature *366*, 276–279.
18. Suzuki, H., Nakayama, T., Yamaguchi, M., and Nishino, T. (2004). cDNA cloning and characterization of two *Dendranthema* × *morifolium* anthocyanin malonyltransferases with different functional activities. Plant Sci. *166*, 89–96.
19. Tohge, T., Nishiyama, Y., Hirai, M.Y., Yano, M., Nakajima, J., Awazuhara, M., Inoue, E., Takahashi, H., Goodenowe, D.B., Kitayama, M., et al. (2005). Functional genomics by integrated analysis of metabolome and transcriptome of Arabidopsis plants over-expressing an MYB transcription factor. Plant J. *42*, 218–235.
20. Luo, J., Butelli, E., Hill, L., Parr, A., Niggeweg, R., Bailey, P., Weisshaar, B., and Martin, C. (2008). AtMYB12 regulates caffeoyl quinic acid and flavonol synthesis in tomato: expression in fruit results in very high levels of both types of polyphenol. Plant J. *56*, 316–326.
21. Mehrtens, F., Kranz, H., Bednarek, P., and Weisshaar, B. (2005). The Arabidopsis transcription factor MYB12 is a flavonol-specific regulator of phenylpropanoid biosynthesis. Plant Physiol. *138*, 1083–1096.
22. Chanoca, A., Kovinich, N., Burkel, B., Stecha, S., Bohorquez-Restrepo, A., Ueda, T., Eliceiri, K.W., Grotewold, E., and Otegui, M.S. (2015). Anthocyanin vacuolar inclusions form by a microautophagy mechanism. Plant Cell *27*, 2545–2559.
23. Poustka, F., Irani, N.G., Feller, A., Lu, Y., Pourcel, L., Frame, K., and Grotewold, E. (2007). A trafficking pathway for anthocyanins overlaps with the endoplasmic reticulum-to-vacuole protein-sorting route in Arabidopsis and contributes to the formation of vacuolar inclusions. Plant Physiol. *145*, 1323–1335.
24. Bae, R.-N., Kim, K.-W., Kim, T.-C., and Lee, S.-K. (2006). Anatomical observations of anthocyanin rich cells in apple skins. HortScience *41*, 733–736.

25. Mol, J., Grotewold, E., and Koes, R. (1998). How genes paint flowers and seeds. Trends Plant Sci. *3*, 212–217.

26. Andersen, Ø.M., and Jordheim, M. (2010). 3.16 - Chemistry of flavonoid-based colors in plants. In Comprehensive Natural Products II, L. Mander, and H.-W. Liu, eds. (Oxford: Elsevier), pp. 547–614.

27. McIlvaine, T.C. (1921). A buffer solution for colorimetric comparision. J. Biol. Chem. *49*, 183–186.

28. Conn, S., Franco, C., and Zhang, W. (2010). Characterization of anthocyanic vacuolar inclusions in *Vitis vinifera* L. cell suspension cultures. Planta *231*, 1343–1360.

29. Jasik, J., and Vancova, B. (1992). Cytological study of anthocyanin production in grapevine (*Vitis vinifera* L.) callus cultures. Acta Bot. Hung. *37*, 251–259.

30. Seidel, T., Golldack, D., and Dietz, K.-J. (2005). Mapping of C-termini of V-ATPase subunits by in vivo-FRET measurements. FEBS Lett. *579*, 4374–4382.

31. Martinière, A., Bassil, E., Jublanc, E., Alcon, C., Reguera, M., Sentenac, H., Blumwald, E., and Paris, N. (2013). In vivo intracellular pH measurements in tobacco and Arabidopsis reveal an unexpected pH gradient in the endomembrane system. Plant Cell *25*, 4028–4043.

32. Gerendás, J., Ratcliffe, R.G., and Sattelmacher, B. (1990). $^{31}$P nuclear magnetic resonance evidence for differences in intracellular pH in the roots of maize seedlings grown with nitrate or ammonium. J. Plant Physiol. *137*, 125–128.

33. Brauer, D., Uknalis, J., Triana, R., and Tu, S.-I. (1997). Effects of external pH and ammonium on vacuolar pH in maize roothair cells. Plant Physiol. Biochem. *35*, 31–39.

34. Trouillas, P., Sancho-García, J.C., De Freitas, V., Gierschner, J., Otyepka, M., and Dangles, O. (2016). Stabilizing and modulating color by copigmentation: insights from theory and experiment. Chem. Rev. *116*, 4937–4982.

35. Fernandes, A., Brás, N.F., Mateus, N., and de Freitas, V. (2015). A study of anthocyanin self-association by NMR spectroscopy. New J. Chem. *39*, 2602–2611.

36. Zhang, Y., Butelli, E., and Martin, C. (2014). Engineering anthocyanin biosynthesis in plants. Curr. Opin. Plant Biol. *19*, 81–90.

37. Andersen, Ø.M., and Jordheim, M. (2006). The anthocyanins. In Flavonoids: Chemistry, Biochemistry and Applications, Ø.M. Andersen, and K.R. Markham, eds. (London: CRC Press), pp. 471–530.

38. Toki, K., Saito, N., Shigihara, A., and Honda, T. (2008). New diacylated delphinidin 3-rutinoside-5-glucosides isolated from the blue-purple flowers of *Browallia speciosa*. Heterocycles *75*, 1503–1509.

39. Pourcel, L., Irani, N.G., Lu, Y., Riedl, K., Schwartz, S., and Grotewold, E. (2010). The formation of anthocyanic vacuolar inclusions in Arabidopsis thaliana and implications for the sequestration of anthocyanin pigments. Mol. Plant *3*, 78–90.

40. Bloor, S.J., and Abrahams, S. (2002). The structure of the major anthocyanin in Arabidopsis thaliana. Phytochemistry *59*, 343–346.

Current Biology, Volume *27*

# Supplemental Information

# Aromatic Decoration Determines the Formation of Anthocyanic Vacuolar Inclusions

**Kalyani Kallam, Ingo Appelhagen, Jie Luo, Nick Albert, Huaibi Zhang, Simon Deroles, Lionel Hill, Kim Findlay, Øyvind M. Andersen, Kevin Davies, and Cathie Martin**

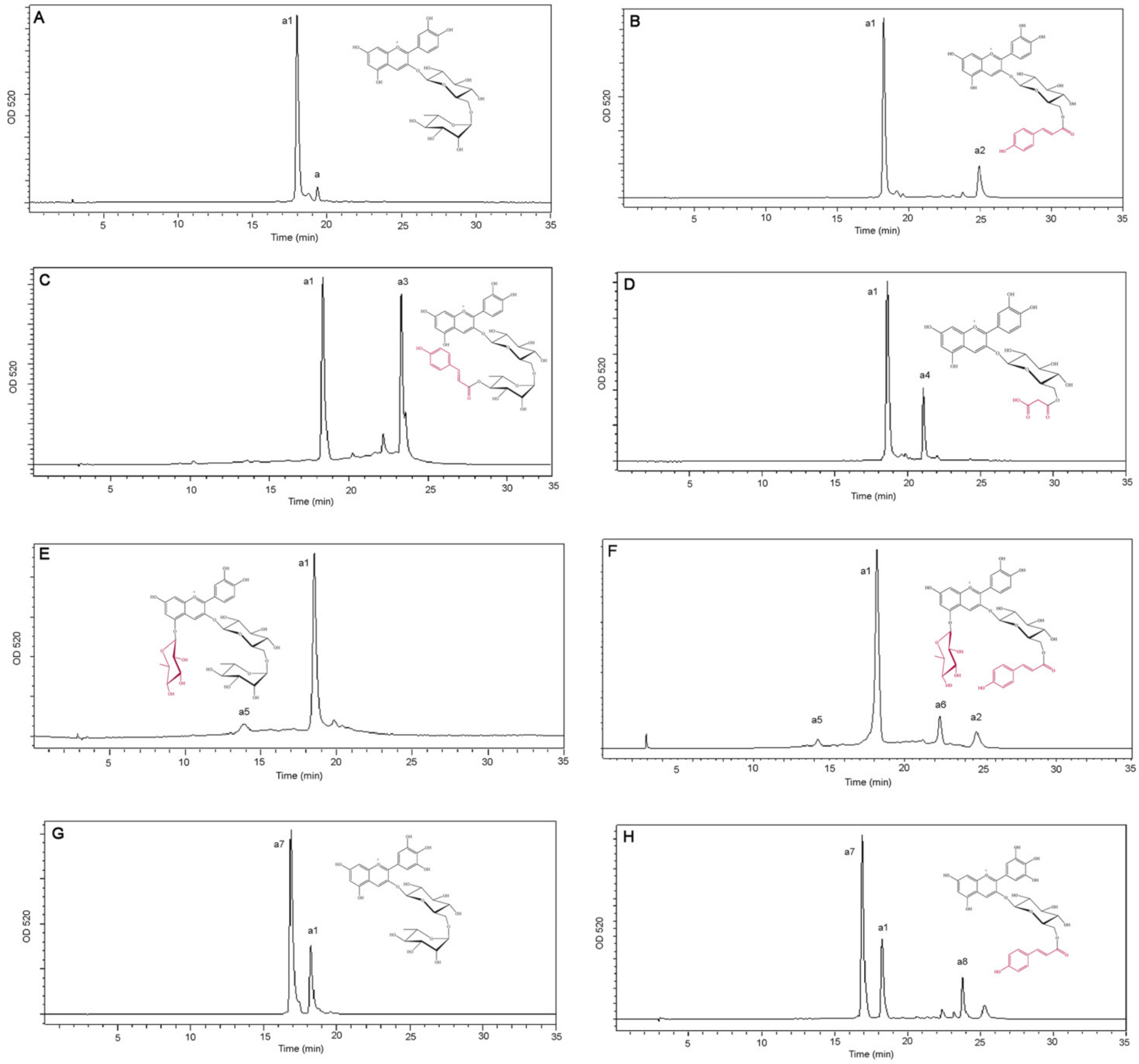


**Figure S1: HPLC separation of anthocyanins in different cell lines (Related to Figure 1 and Figure 2)**

HPLC chromatograms of leaf extracts in methanol and recorded at 520 nm (**A**) *Del/Ros1* (**B**) *Del/Ros1/At3AT* (**C**) *Del/Ros1/Sl3AT* (**D**) *Del/Ros1/Ci3MAT* (**E**) *Del/Ros1/At5GT* (**F**) *Del/Ros1/At3AT/At5GT* (**G**) *Del/Ros1/PhF3'5'H* (**H**) *Del/Ros1/At3AT/PhF3'5'H*. The methanol extracts of anthocyanins were run on the Surveyor HPLC attached to DecaXPplus ion trap MS (Thermo) and separation was on a 100×2mm 3μm Luna C18(2) column (Phenomenex). Anthocyanin peaks are labelled with numbered alphabets and identity of the new compounds are provided next to the peaks. a=pelargonidin 3-*O*-rutinoside; a1=cyanidin 3-*O*-rutinoside; a2= cyanidin 3-*O*-(6''-*O*-(coumaroyl) glucoside); a3=cyanidin 3-*O*-(4'''-*O*-(coumaroyl) rutinoside); a4=cyanidin 3-*O*-(6''-*O*-(malonyl) glucoside); a5=cyanidin 3-*O*-rutinoside, 5-*O*-glucoside; a6=cyanidin 3-*O*-(6''-*O*-(coumaroyl) glucoside)-5-*O*-glucoside; a7=delphinidin 3-*O*-rutinoside; a8=delphinidin 3-*O*-(6''-*O*-(coumaroyl) glucoside)

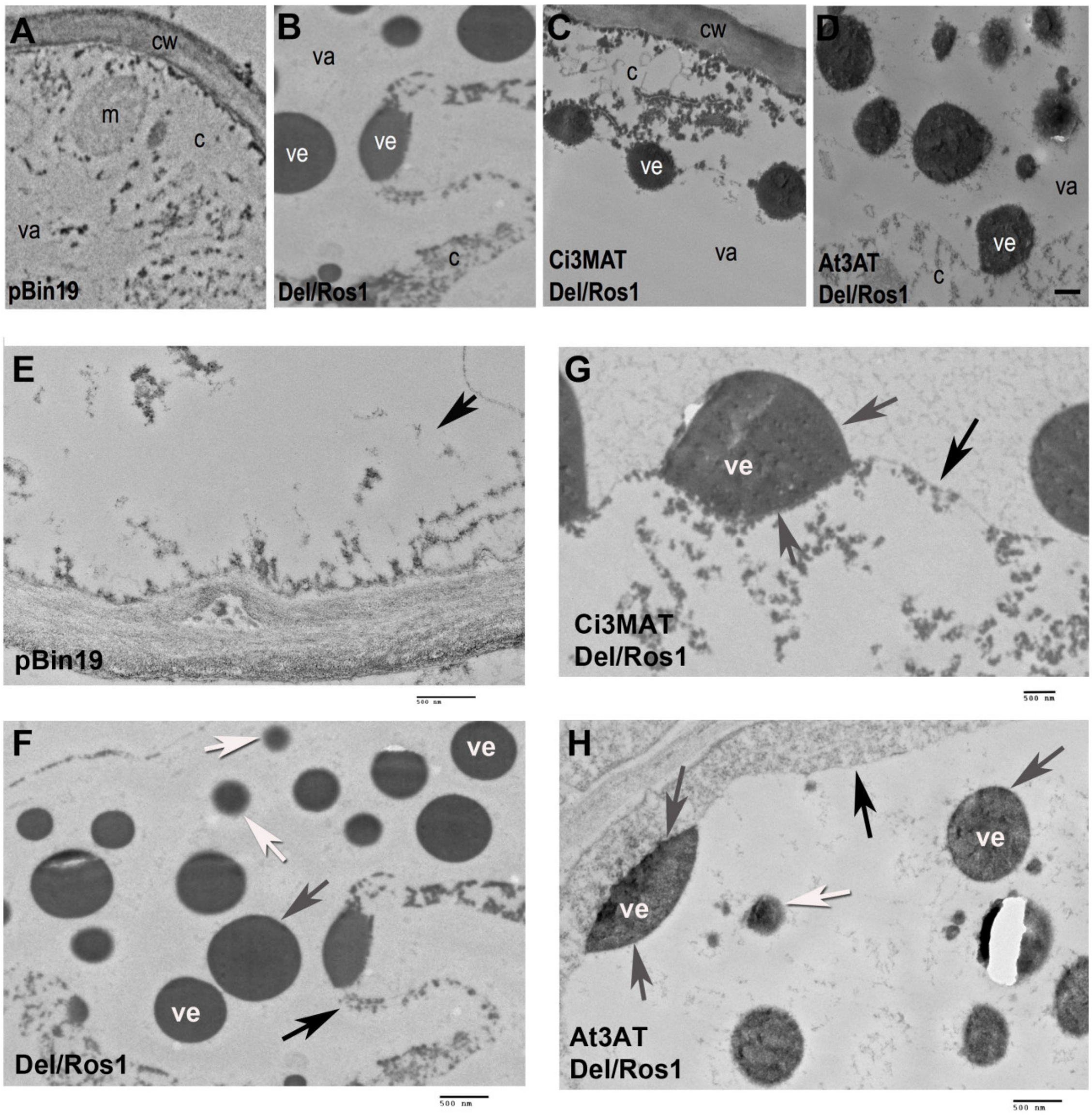


**Figure S2. Transmission electron micrographs of callus culture cells (Related to Figure 2)**

(**A**) Wild type tobacco cell transformed with an empty pBin19 binary vector alone. (**B**) *Del/Ros1* making cyanidin 3-*O*-rutinoside only, producing no AVIs, (**C**) *Del/Ros1/Ci3MAT* making cyanidin 3-*O*-rutinoside and cyanidin 3-*O*-(6''-*O*-(malonyl) glucoside), producing no AVIs. (**D**) *Del/Ros1/At3AT* making cyanidin 3-*O*-rutinoside and cyanidin 3-*O*-(6''-*O*-(coumaroyl) glucoside) and producing AVIs. c= cytoplasm, va = vacuole, cw = cell wall, ve = anthocyanin-filled vesicle, m = mitochondrion. Scale bar indicates 0.5 µm. (**E**) Higher magnification image of wild type tobacco cell transformed with an empty pBin19 binary vector alone (**F**) Higher magnification image of *Del/Ros1* making cyanidin 3-*O*-rutinoside only, producing no AVIs (**G**) Higher magnification image of *Del/Ros1/Ci3MAT* making cyanidin 3-*O*-rutinoside and cyanidin 3-*O*-(malonyl) glucoside, producing no AVIs (**H**) *Del/Ros1/At3AT* making cyanidin 3-*O*-rutinoside and cyanidin 3-*O*-(coumaroyl) glucoside and producing AVIs. (**E-H**) ve shows anthocyanin filled vesicle, black arrows indicate tonoplast, grey arrows indicate membranes around vesicles and white arrows indicate vacuolar vesicles around which membranes are disintegrating; scale bars show 500 nm.

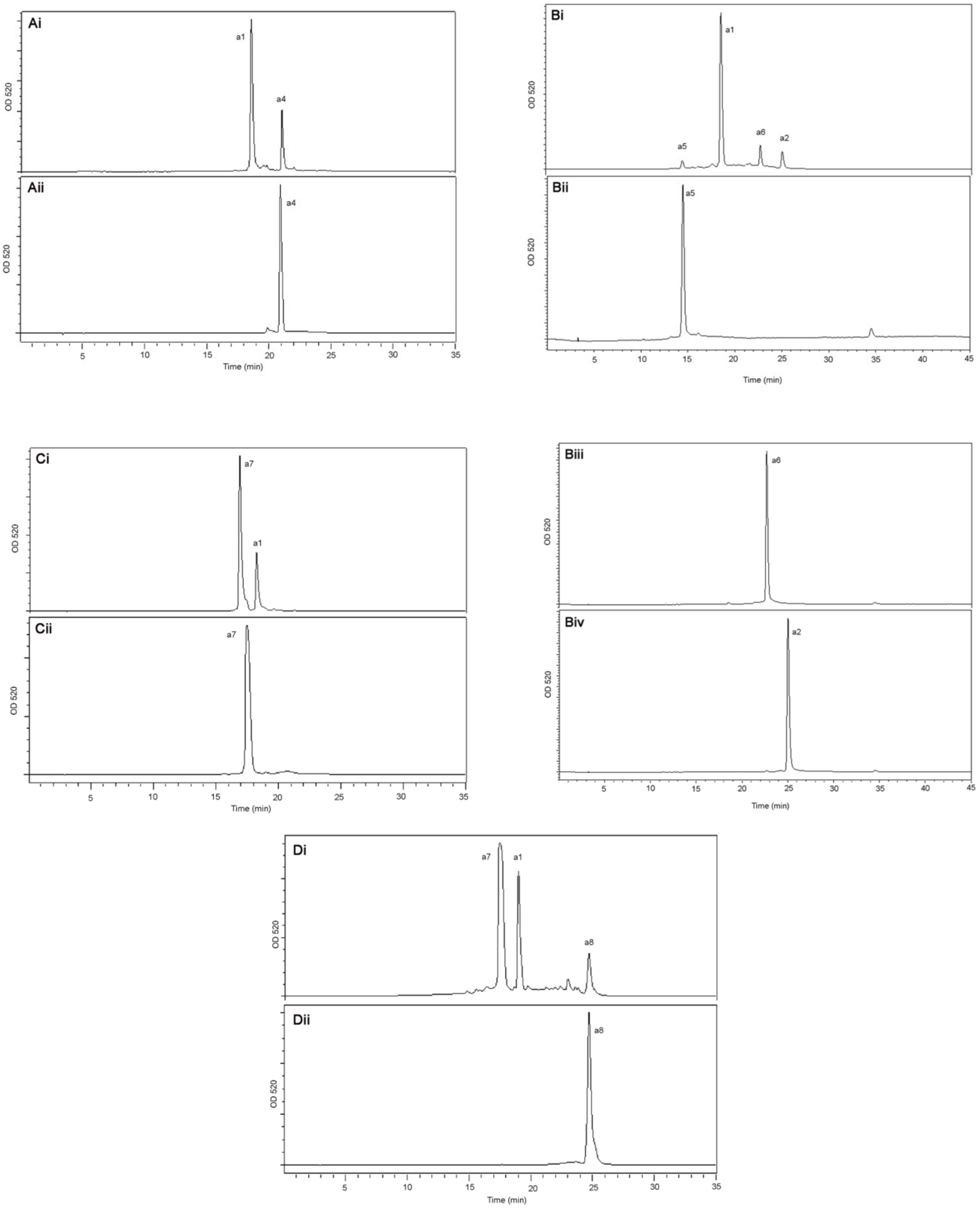


**Figure S3. Analytical HPLC analysis of anthocyanins purified from tobacco (Related to Figure 3)**

Chromatograms of purified anthocyanins in methanol as studied through HPLC and recorded at 520 nm (**Ai**) Total anthocyanins from leaf extracts of *Del/Ros1/Ci3MAT* and (**Aii**) purified anthocyanin, peak-a1; cyanidin 3-*O*-rutinoside and peak-a4; cyanidin 3-*O*-(malonyl) glucoside (**Bi**) Extracts of total anthocyanins from *Del/Ros1/At3AT/At5GT* line and (**Bii, Biii, Biv**) purified anthocyanins, peak-a5; cyanidin 3-*O*-rutinoside-5-*O*-glucoside, peak-a6; cyanidin 3-*O*-(6''-*O*-(coumaroyl) glucoside)-5-*O*-glucoside, peak-a2; cyanidin 3-*O*-(6''-*O*-(coumaroyl) glucoside) (**Ci**) HPLC chromatogram of total anthocyanins from leaf extracts of *Del/Ros1/PhF3'5'H* and (**Cii**) purified anthocyanin, peak-a1; cyanidin 3-*O*-rutinoside and peak-a7; delphinidin 3-*O*-rutinoside (**Di**) HPLC chromatogram of total anthocyanins from leaf extracts of *Del/Ros1/PhF3'5'H/At3AT* and (**Dii**) purified anthocyanin, peak-a1; cyanidin 3-*O*-rutinoside, peak-a7; delphinidin 3-*O*-rutinoside, peak-a8; delphinidin 3-*O*-(6''-*O*-(coumaroyl) glucoside). Further confirmation of purity of anthocyanins was achieved by mass fragmentation (LC-MS) and chromatogram studies at 280 nm.

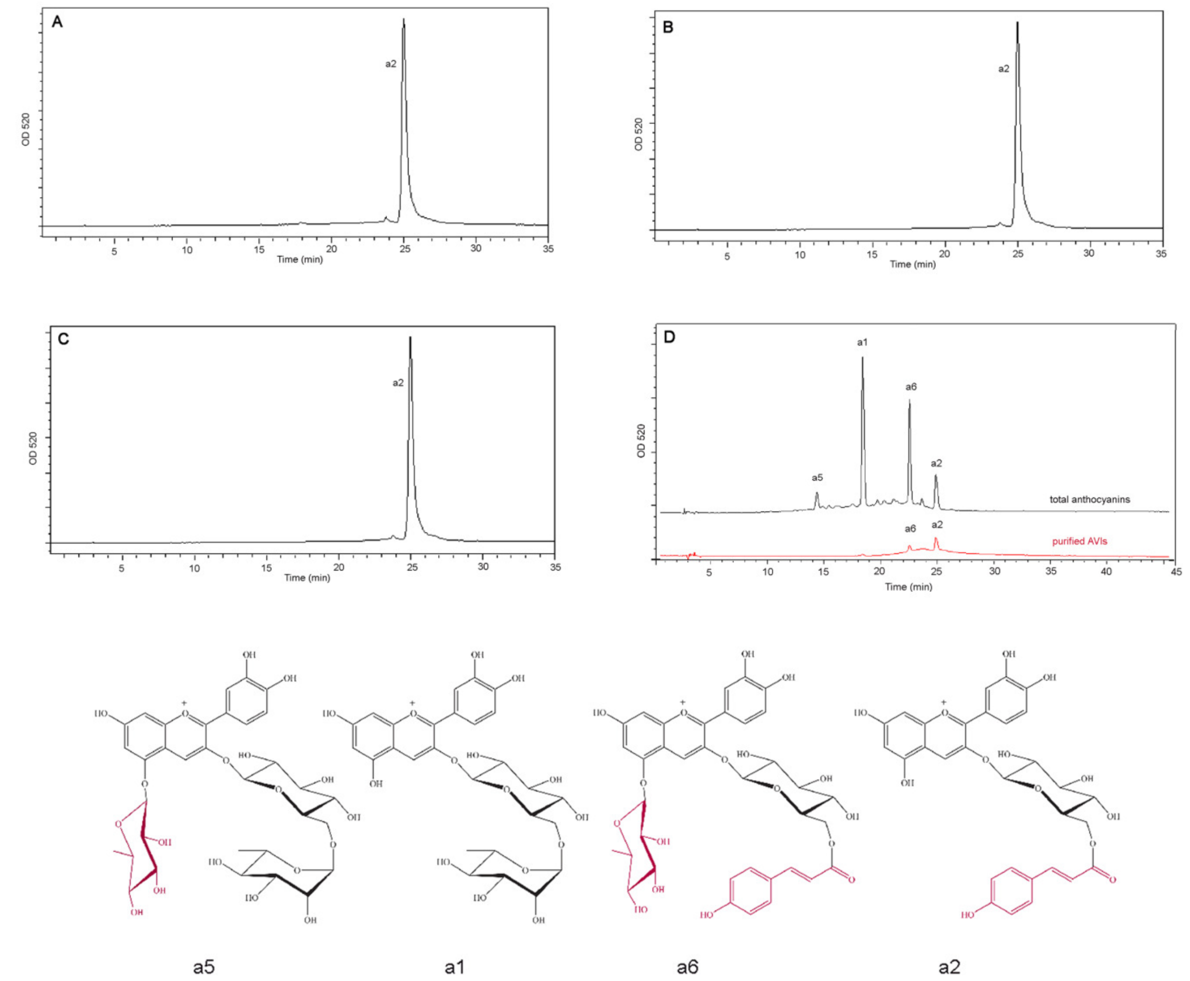


**Figure S4. Purification and analysis of *in vitro* anthocyanin precipitates (Related to Figure 3)**

**(A-C)** A sample of purified cyanidin 3-*O*-(6''-*O*-(coumaroyl)glucoside) in 80% methanol was separated by HPLC (**A**). Cyanidin 3-*O*-(6''-*O*-(coumaroyl)glucoside) was added to 0.4 M sodium acetate buffer at pH 4.5 (**B**) or McIlvaine's buffer at pH 7.0 (**C**) and *in vitro* precipitates were purified by centrifugation and washing in buffer, then re-dissolved in 80% methanol and separated by HPLC. The peaks from the precipitates ran at exactly the same position (just over 25 minutes) as the cyanidin 3-*O*-(6''-*O*-(coumaroyl)glucoside) in solution.
**(D)** Purification of precipitates from anthocyanin mixtures from *Del/Ros/At3AT/At5GT* line. HPLC profiles of total anthocyanins (top in black) and anthocyanins extracted from precipitates formed *in vitro* by addition of 0.4 M sodium acetate buffer, pH 4.5 (bottom in red). Peak-a5; cyanidin 3-*O*-glucoside-5-*O*-glucoside, peak-a1; cyanidin 3-*O*-rutinoside. Cyanidin 3-*O*-(6''-*O*-(coumaroyl) glucoside)-5-*O*-glucoside (peak a6) and cyanidin 3-*O*-(6''-*O*-(coumaroyl)glucoside) (peak a2) were detected in AVIs with the major anthocyanin being cyanidin 3-*O*-(6''-*O*-(coumaroyl)glucoside) (peak a2). Chemical structures of the anthocyanins corresponding to different peaks (a1, a2, a5 and a6) are shown below the HPLC profiles.

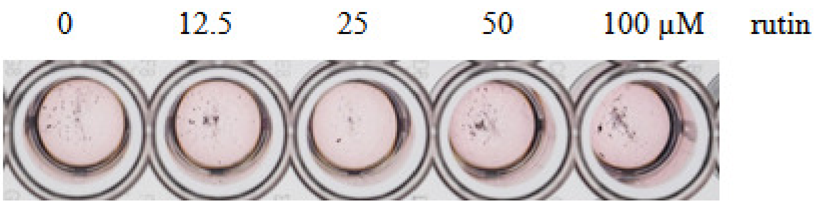


**Figure S5. Effects of flavonols on anthocyanin precipitation *in vitro* (Related to Figure 2)**

*In vitro* precipitation of cyanidin 3-*O*-(6''-*O*-(coumaroyl)glucoside) from tobacco in 0.1 M sodium citrate buffer at pH 4.5 with increasing concentrations of the flavonol glycoside, rutin, added prior to adding the anthocyanin at 50 µM.

| Transgenic line | Peak label | Rt (min) | ESI-MS (m/z) | MS/MS fragments | Compound |
|---|---|---|---|---|---|
| Del/Ros1 | a1<br>a | 18.65<br>19.9 | 595<br>579.1 | 449 [Cy+Glc]$^+$ 287 [Cy]$^+$<br>433 [Pg+Glc]$^+$ 271.3 [Pg]$^+$ | cyanidin 3-*O*- rutinoside<br>pelargonidin 3-*O*-rutinoside |
| Del/Ros1/ At3AT | a1<br>a2 | 18.48<br>25.04 | 595<br>595 | 449 [Cy+Glc]$^+$ 287 [Cy]$^+$<br>449 [Cy+Glc]$^+$ 287 [Cy]$^+$ | cyanidin 3-*O*- rutinoside<br>cyanidin 3-*O*-(6′′-*O*- (coumaroyl) glucoside) |
| Del/Ros1/ Sl3AT | a1<br>a3 | 18.37<br>23.33 | 595.1<br>741.2 | 449 [Cy+Glc]$^+$ 287 [Cy]$^+$<br>594.9 [Cy+Rha]$^+$ 449 [Cy+Glc]$^+$ 287 [Cy]$^+$ | cyanidin 3-*O*- rutinoside<br>cyanidin 3-*O*-(6′′-*O*- (coumaroyl) rutinoside) |
| Del/Ros1/ Ch3MAT | a1<br>a4 | 17.97<br>20.6 | 595<br>535 | 449 [Cy+Glc]$^+$ 287 [Cy]$^+$<br>449 [Cy+Glc]$^+$ 287 [Cy]$^+$ | cyanidin 3-*O*- rutinoside<br>cyanidin 3-*O*-(6′′ -*O*-(malonyl) glucoside ) |
| Del/Ros1/ At5GT | a5<br><br>a1 | 13.9<br><br>18.52 | 751.1<br><br>595 | 595 [Cy+Rha]$^+$ 449 [Cy+Glc]$^+$ 287 [Cy]$^+$<br>449 [Cy+Glc]$^+$ 287 [Cy]$^+$ | cyanidin 3-*O*- rutinoside, 5- *O*-glucoside<br><br>cyanidin 3-*O*- rutinoside |
| Del/Ros1/ At3AT/ At5GT | a1<br>a5<br><br>a6<br><br>a2 | 18.45<br>14.26<br><br>22.31<br><br>24.75 | 595<br>751.1<br><br>757<br><br>595 | 449 [Cy+Glc]$^+$ 287 [Cy]$^+$<br>595 [Cy+Rha]$^+$ 449 [Cy+Glc]$^+$ 287 [Cy]$^+$<br>595 [Cy+Glc+Cou]$^+$ 449 [Cy+Glc]$^+$ 287 [Cy]$^+$<br>449 [Cy+Glc]$^+$ 287 [Cy]$^+$ | cyanidin 3-*O*- rutinoside<br>cyanidin 3-*O*- rutinoside-5-glucoside<br><br>cyanidin 3-*O*-(6′′-*O*- (coumaroyl) glucoside)-5-*O*-glucoside<br>cyanidin 3-*O*-(6''-*O*-(coumaroyl) glucoside) |
| Del/Ros1/ PhF3'5'H | a7<br>a1 | 16.78<br>18.19 | 611.1<br>595 | 464.97 [Dp+Glc]$^+$ 303.1 [Dp]$^+$<br>449 [Cy+Glc]$^+$ 287 [Cy]$^+$ | delphinidin 3-*O*-rutinoside<br>cyanidin 3-*O*- rutinoside |
| Del/Ros1/ PhF3'5'H/ At3AT | a7<br>a1<br>a8<br>a2 | 16.9<br>18.27<br>23.79<br>25.29 | 611.1<br>595<br>611.14<br>595 | 464.97 [Dp+Glc]$^+$ 303.1 [Dp]$^+$<br>449 [Cy+Glc]$^+$ 287 [Cy]$^+$<br>464.97 [Dp+Glc]$^+$ 303.1 [Dp]$^+$<br>449 [Cy+Glc]$^+$ 287 [Cy]$^+$ | delphinidin 3-*O*-rutinoside<br>cyanidin 3-*O*- rutinoside<br>delphinidin 3-*O*-(6′′ -*O*-(coumaroyl) glucoside)<br>cyanidin 3-*O*-(6′′ -*O*-(coumaroyl) glucoside) |

**Table S1. Characterisation of anthocyanins engineered in tobacco (Related to Figure 3 and Figure S1)**

Characteristics of anthocyanins from Figure S1; their molecular ions and corresponding fragments together with the identity of each compound are tabulated. ESI-MS; Electro-spray ionization mass spectra, *m/z*; molecular mass of compound. The molecular mass and fragmentation patterns of cyanidin 3-*O*-rutinoside, 5-*O*-glucoside and cyanidin 3-*O*-(6''-*O*-(coumaroyl)glucoside)-5-*O*-glucoside being the same, further NMR confirmation of cyanidin 3-*O*-(6''-*O*-(coumaroyl)glucoside)-5-*O*-glucoside was performed with purified compounds (data not provided). Rt, retention time; Cy; cyanidin, Dp; delphinidin, Pg; pelargonidin, Rha; rhamnose, Glc; glucose

| Peak | ESI-MS (m/z) | Mass accuracy (ppm) | Formula | Compound |
|---|---|---|---|---|
| 1 | [M]$^+$ 1337.3655 | 3.07 | $C_{59}H_{69}O_{35}^+$ | Violdelphin+Glc |
| 2 | [M]$^+$ 1175.3099 | 1.11 | $C_{53}H_{59}O_{30}^+$ | Violdelphin |
| 3 | [M]$^+$ 1029.2519 | 1.17 | $C_{47}H_{49}O_{26}^+$ | Violdelphin-Rha |
| 4 | [M]$^+$ 1901.5198<br>[M+K]$^{2+}$ 970.2327 | 5.52<br>3.71 | $C_{85}H_{97}O_{49}^+$ | Cyanodelphin |

**Table S2. Characterisation of anthocyanins in delphinium flowers (Related to Figure 5)**

IT-ToF peak identification of violdelphin and cyanodelphin are tabulated. ESI-MS; Electro-spray ionization mass spectra, *m/z*; molecular mass of compound.

**Supplemental Experimental Procedures**

**Generation of Plant Material and Growth Conditions**

A full length gene, encoding anthocyanin UDP-glucoronosyl/UDP-glucosyl transferase (GeneBank accession AT4G14090 [S1]), a full length cDNA of At1g03940 encoding an anthocyanidin 3-glucoside acyl CoA transferase (At3AT1 [S2]), a full length cDNA (EU979541) encoding anthocyanin 3-rutinoside hydroxycinnamoyl CoA transferase from Solanum lycopersicum cv. MicroTom (Sl3AT1 [S3, S4]), a cDNA encoding flavonoid 3'5' hydroxylase from Petunia hybrida (PhF3'5'H [S5]; CAA80265), and a cDNA from Chrysanthemum indicum, encoding anthocyanidin 3-*O*-glucoside-malonyltransferase Ci3MaT1 (GeneBank accession AY298809 [S6]), amplified from cDNA of dark red (maroon) chrysanthemum inflorescence were cloned into pJAM1502 [S2]. Gene specific primers having Gateway™ recombination sequences; attB1 and attB2 used to amplify each gene sequence are given at the end of this section. Wild type and transgenic tobacco plants were grown at 23- 25°C under 16 h light/8 h dark cycle. Callus from young and healthy transgenic leaf material of genotyped plants was induced on callus induction medium (MS agar (0.8% w/v) supplemented with kanamycin 100 $mgL^{-1}$, kinetin 0.5 $mgL^{-1}$, 2,4 D 1.0 $mgL^{-1}$ and NAA 0.5 $mgL^{-1}$). The plates were incubated in a growth room at 23-25°C under 16 h light/8 h dark or in complete darkness at 23-25°C. Callus formation was better when maintained in darkness, and this condition was applied for all the cultures. Sub-culturing was performed every 15 d for 45 d, until sufficient callus was produced for suspension culture formation.

**Anthocyanin extraction and semi-purification**

Flower petals from which anthocyanins were to be extracted were ground to a fine powder. Extraction solvent, (70% methanol with 0.1 % HCl, 40 $\mu Lmg^{-1}$) was added to the samples, which were vortexed thoroughly and sonicated in a water bath for 10 min. Extracts were processed immediately by centrifugation at 12,000 rpm for 10 min at 4°C. Supernatants were collected and stored at -20°C before being analysed. Anthocyanins from finely ground leaf material were extracted using acidified water (0.01% HCl). Two steps of extraction were followed by addition of an equal amount of chloroform at 4°C to remove the chlorophyll. The supernatant was collected the following day and rotary evaporated under vacuum at 30-40°C until any residual chloroform had evaporated. Anthocyanins in acidified water were purified [S7] by passing through a preconditioned C18 mini cartridge (Sep-Pak Cartridge 500 mg sorbent, Waters Chromatography®). The C18 cartridge was initially conditioned by passing over two column volumes of methanol followed by three column volumes of acidified deionized milliQ water to remove the remaining methanol. Each sample was loaded onto the column until excess colour had passed through the cartridge. The cartridge was washed with two column volumes of acidified water to remove compounds not adsorbed (sugars, acids) followed by two column volumes of ethyl acetate to remove polyphenolic compounds such as phenolic acids and flavonols. Anthocyanin pigments were eluted with acidified methanol. Methanol was removed from the extract in a rotary evaporator at 40°C under vacuum and the anthocyanins were re-dissolved in acidified milliQ water and stored at -20°C until further use.

**Preparative HPLC purification**

Anthocyanins were purified using a Gilson Preparative High Pressure Liquid Chromatography (Prep HPLC) System. Partially purified and filtered extracts (filtered using a 0.2 µm polyfluortetraethylene filter, Sartorius) were applied onto a 250 x 21.2 mm C18 column (Phenomenex) fitted with a guard column (Phenomenex Prodigy). The column was pre-equilibrated with water containing 0.5% triflouroacetic acid (TFA). The sample was injected onto the column, developed by a wash with 96% solvent A (0.5% TFA in water) and 4% solvent B (0.5% TFA in 50% acetonitrile (ACN)). Anthocyanins were eluted with a linear gradient of increasing solvent B at a flow rate of 1 mL $min^{-1}$: from 20% to 40% over 10 min, 40% to 60% over 5 min, 60% to 80% over 13 min, 80% to 100% over 7 min and 100% for 10 min. The elution products were monitored with a UV detector. This protocol was developed for separation of all the compounds. Fractions were collected and residual acetonitrile in the fractions was removed using a rotary evaporator. Acidified water was added to the samples and freeze dried. Freeze dried anthocyanin samples or concentrated samples after rotary evaporation were stored at -80°C in an air tight container or dissolved in the desired solvent for further studies.

**HPLC, LC-MS analysis and quantification of anthocyanins**

Anthocyanin extracts were run on a 250 x 4.6 mm internal diameter Spherisorb® 5 µm C18 column using Waters HPLC system (Waters, http://www.waters.com). The column was initially equilibrated with 96% solvent A (0.5% TFA) and 4% solvent B (0.5% TFA in 50% ACN) and eluted with a gradient of increasing solvent B at a flow rate of 1 mL $min^{-1}$. After injection the column was washed with 96% solvent A followed by a linear gradient of solvent B from 4% to 20% in 5 min, 20% to 40% in 10 min, 40% to 60% in 5 min, 60% to 20% in

10 min and 20% to 4% in 5 min. The elution products were monitored with a photo diode array (PDA) detector over the range 200–600 nm. Anthocyanin contents were analysed based on the peaks obtained at various retention times compared to controls and quantified based on the peak areas at 520 nm in the chromatograms. Commercial anthocyanins: cyanidin 3-*O*-rutinoside and cyanidin 3-*O*-glucoside (Extrasynthase, France) were used as standards. Standards (5 mg) were dissolved in methanol with 0.1% HCl and stored at -80°C. The concentration of anthocyanins in the samples was calculated by measuring the absorbance at 520 nm using a Spectra max 340PC384 (Molecular devices) spectrophotometer [S8]. Readings were analysed using Softmax(R) Pro software version 4.8. Purified anthocyanins and their structural decorations were always confirmed by LC/MS using a Thermo Finnigan Surveyor HPLC system (Thermo Scientific, http://www.thermo.com) equipped with a diode array (PDA) detector and a Deca XP plus ion trap mass spectrometer (Thermo Scientific), prior to undertaking new assays. Anthocyanin masses and fragmentation patterns were analysed using Quant browser.

Anthocyanin samples from delphinium sepals were run on a Shimadzu Nexera LC system attached to an IT ToF mass spectrometer. Separation was on a 100×2.1 mm 2.6 µ Kinetex EVO-C18 column (Phenomenex), using a linear gradient of acetonitrile versus 0.1% formic acid from 2% to 10% acetonitrile in 2.5 min, 10% to 30% in 10 min, 30% to 90% in 5.8 min, from 90% to 2% in 4.3 min, with a flow rate of 0.5mL $min^{-1}$. Positive electrospray MS spectra from m/z 220-2000 were collected with a maximum ion accumulation time of 20 msec and an automatic sensitivity control target of 70% optimum base peak intensity.

## Protoplast and AVI isolation

A protocol for protoplast isolation was adapted from several published protocols. Leaf material or flower material was cut into small pieces in protoplast buffer (0.7 M mannitol, 0.25 mM MES, 1% (w/v) cellulase, 0.25% (w/v) macerozyme, 0.5% (w/v) driselase; pH 5.5) and incubated at 30°C for 4-6 h in dark with gentle shaking (30 rpm). The solution was strained through two layers of Mira cloth and a layer of 50 µm cheese cloth. The filtrate was centrifuged at 4°C for 10 min at 100 rpm. The pellet was washed in protoplast buffer without enzymes and suspended in a small amount of protoplast buffer. Ten times the volume of suspended pellet of 0.7 M sucrose was layered on top of the pellet, centrifuged for 15 min at 100 rpm, 4°C. Intact and healthy protoplasts are collected on top of the sucrose solution. Protoplasts were washed twice with the buffer and suspended in a small amount of buffer and stored at -20°C. Freeze-thawed protoplasts were sonicated to break them and layered on a Percoll gradient (obtained by sequential layering of 80%/50%/30%/10% Percoll with protoplast buffer) and centrifuged at 100 rpm for 10-15 min. AVIs were collected from the bottom of the tube prior to analysis.

## Confocal microscopy

Images were taken with Leica SP5II and SP8X confocal microscopes equipped with HCX APO L U-V-I 63.0 x 0.90 UV and HC PL APO CS2 63x/1.20 water immersion objectives (both from Leica Microsystems GmbH). Fluorescence was detected by hybrid detectors unless stated otherwise. Emission spectra of anthocyanins were recorded with 3 nm step size and 10 nm bandwidth after excitation at 561 nm with a diode-pumped solid-state laser. Anthocyanins were subsequently detected between 600-650 nm in leaf tissues to avoid detection of chlorophyll auto-fluorescence, and between 665-730 nm in callus cells without chloroplasts. pH measurements in vacuoles were performed as described by Siedel et al., (2005) [S9] with the following modifications. Leaf disks were incubated overnight in 1xMS medium with 20µM of the non-fluorescent dye 6-carboxyfluorescein diacetate (6-CFDA, Sigma-Aldrich) and 0.35‰ (v/v) Triton X-100 (Sigma-Aldrich). Non-specific vacuolar esterases cleave 6-CFDA and release the fluorescent dye 6-carboxyfluorescein (6-CF), which has a pH-dependent ratio of emission intensities at different excitation wavelengths. 6-CF was excited sequentially using the 458 nm and the 488 nm line of an argon laser and detected in the range of 500-530 nm to avoid crosstalk with anthocyanins. A HC PL APO CS2 20x/0.75 air objective (Leica Microsystems GmbH) was used and images were acquired in 12 bit resolution to maximise the dynamic range. Calibrations were performed with a 6-CF standard (Sigma-Aldrich) in buffers with pH-values from pH 4.65 to 7.0 (1/20 dilutions of a 10 mM 6-CF stock in ethanol), whereby the intensities of the laser lines were adjusted to an emission ratio of about 1.0 at pH 6. Fluorescence from 20 epidermal cells was measured and pH values were calculated from a linear regression curve of the 6-CF reference measurements. For membrane stains, cells were incubated for 16 to 20 h in 4 µM FM1-43 (Thermo Scientific) in 1xMS medium containing 3% (w/v) sucrose. Tobacco cells were incubated in 24-well plates and the dye infiltrated into delphinium sepals with a syringe. FM1-43 fluorescence was detected by PMT2 (Leica SP5) in the range of 530-590 nm and excited using the 476 nm line of an argon ion laser. Anthocyanins were detected as described before. Both fluorophores were recorded sequentially, line by line.

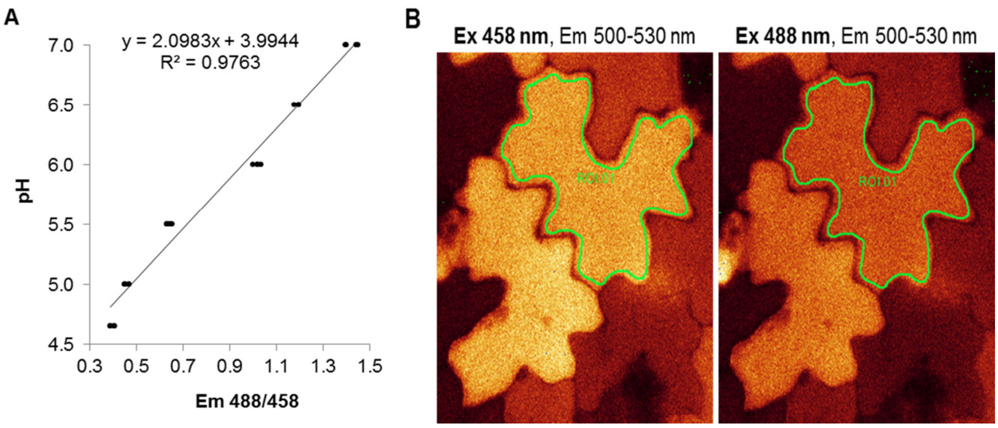


***In vivo* pH measurements**: (A) 6-CF calibration curve showing the ratio of emissions intensities, which were detected after excitation at 458 nm and 488 nm. pH values in vacuoles of epidermal cells were calculated as pH = (($Em_{488nm}/Em_{458nm}$) * 2.0983) + 3.9944. (B) Representative images of 6-CF fluorescence in epidermal cells after excitation at 458 nm (left image) and 488 nm (right image). Regions of interest (ROI, shown in green) were marked to calculate pH values for single cells.

**Transmission electron microscopy**

Samples were fixed for 1 hour by the addition of glutaraldehyde to the cell culture to give a 2.5% final concentration then pre-embedded in low-gelling temperature agarose before further fixation in 2.5% (v/v) glutaraldehyde in 0.05 M sodium cacodylate, pH 7.3, overnight. Samples were dehydrated through an ethanol series then gradually infiltrated with LR White resin (London Resin Company, Reading, Berkshire) before polymerisation at 60°C. Samples were sectioned using a Leica UC6ultramicrotome (Leica, Milton Keynes) to give ultrathin sections of approximately 90nm. Sections were stained with 2% (w/v) uranyl acetate for 1hr and 1% (w/v) lead citrate for 1 minute, washed in distilled water and air dried. Grids were viewed in a FEI Tecnai 20 transmission electron microscope (FEI UK Ltd, Cambridge, UK) at 200 kV and imaged using an AMT XR60 digital camera (Deben, Bury St Edmunds, UK) to record TIF files.

**Sequences of Gateway primers used for cloning**

| Primer name | Primer sequence |
|---|---|
| B1Ptf35hF | 5' GGGGACAAGTTTGTACAAAAAAGCAGGCTGGACC ATGATGCTACTTACTGAGCTTG 3' |
| B2 Ptf35hR | 5' GGGGACCACTTTGTACAAGAAAGCTGGGTCCT ATGGTACATAAACATCCAATTGTAA 3' |
| Ci3MaT1B1 F | 5' GGGGACAAGTTTGTACAAAAAAGCAGGCTGGAT GGCTTCCAATTCCATTGTTA 3' |
| Ci3MaT1B2 R | 5' GGGGACCACTTTGTACAAGAAAGCTGGGTCT TATATCTCACTCTCTAATCCG 3' |
| B1AAT | 5' GGGGACAAGTTTGTACAAAAAAGCAGGCTGG ATGGTGG CTCATCTTCAACCT 3' |
| B2AAT | 5' GGGGACCACTTTGTACAAGAAAGCTGGGTCCGTTGCGAAT TTCTTGATCCC 3' |
| B1SAT | 5' GGGGACAAGTTTGTACAAAAAAGCAGGCTGGATGAGCC AAATTACAACACAAAA 3' |
| B2SAT | 5' GGGGACCACTTTGTACAAGAAAGCTGGGTCCTTTGGCACAT AACTAAACTC 3' |
| AtGF | 5' GGGGACAAGTTTGTACAAAAAAGCAGGCTGGAT GGCCACTTCCGTCAATGG 3' |
| AtGR | 5' GGGGACCACTTTGTACAAGAAAGCTGGGTCCTAC TCATCCTCGTCCACAAA 3' |

## Supplemental References


S1. Tohge, T., Nishiyama, Y., Hirai, M.Y., Yano, M., Nakajima, J.-i., Awazuhara, M., Inoue, E., Takahashi, H., Goodenowe, D.B., Kitayama, M., et al. (2005). Functional genomics by integrated analysis of metabolome and transcriptome of Arabidopsis plants over-expressing an MYB transcription factor. The Plant Journal *42*, 218-235.

S2. Luo, J., Nishiyama, Y., Fuell, C., Taguchi, G., Elliott, K., Hill, L., Tanaka, Y., Kitayama, M., Yamazaki, M., Bailey, P., et al. (2007). Convergent evolution in the BAHD family of acyl transferases: identification and characterization of anthocyanin acyl transferases from Arabidopsis thaliana. The Plant Journal *50*, 678-695.

S3. Tohge, T., Zhang, Y., Peterek, S., Matros, A., Rallapalli, G., Tandrón, Y.A., Butelli, E., Kallam, K., Hertkorn, N., Mock, H.-P., et al. (2015). Ectopic expression of snapdragon transcription factors facilitates the identification of genes encoding enzymes of anthocyanin decoration in tomato. The Plant Journal *83*, 686-704.

S4. Butelli, E., Titta, L., Giorgio, M., Mock, H.P., Matros, A., Peterek, S., Schijlen, E.G., Hall, R.D., Bovy, A.G., Luo, J., et al. (2008). Enrichment of tomato fruit with health-promoting anthocyanins by expression of select transcription factors. Nature Biotechnology *26*, 1301-1308.

S5. Holton, T.A., Brugliera, F., Lester, D.R., Tanaka, Y., Hyland, C.D., Menting, J.G.T., Lu, C.-Y., Farcy, E., Stevenson, T.W., and Cornish, E.C. (1993). Cloning and expression of cytochrome P450 genes controlling flower colour. Nature *366*, 276-279.

S6. Suzuki, H., Nakayama, T., Yamaguchi, M.A., and Nishino, T. (2004). cDNA cloning and characterization of two Dendranthema x morifolium anthocyanin malonyltransferases with different functional activities. Plant Science *166*, 89-96.

S7. Rodriguez-Saona, L.E., and Wrolstad, R.E. (2001). Extraction, Isolation, and Purification of Anthocyanins. In Current Protocols in Food Analytical Chemistry. (John Wiley & Sons, Inc.).

S8. Giusti, M.M., and Wrolstad, R.E. (2001). Characterization and Measurement of Anthocyanins by UV-Visible Spectroscopy. In Current Protocols in Food Analytical Chemistry. (John Wiley & Sons, Inc.).

S9. Seidel, T., Golldack, D., and Dietz, K.-J. (2005). Mapping of C-termini of V-ATPase subunits by in vivo-FRET measurements. FEBS Letters *579*, 4374-4382.
